# Quantitative proteomic analyses reveal that GPX4 downregulation during myocardial infarction contributes to ferroptosis in cardiomyocytes

**DOI:** 10.1038/s41419-019-2061-8

**Published:** 2019-11-04

**Authors:** Tae-Jun Park, Jei Hyoung Park, Ga Seul Lee, Ji-Yoon Lee, Ji Hye Shin, Min Wook Kim, Yong Sook Kim, Jeong-Yoon Kim, Kyoung-Jin Oh, Baek-Soo Han, Won-Kon Kim, Youngkeun Ahn, Jeong Hee Moon, Jaewhan Song, Kwang-Hee Bae, Do Han Kim, Eun-Woo Lee, Sang Chul Lee

**Affiliations:** 1 Metabolic Regulation Research Center, Korea Research Institute of Bioscience and Biotechnology (KRIBB), Daejeon, 34141 Korea; 2 Department of Microbiology and Molecular Biology, College of Bioscience and Biotechnology, Chungnam National University, Daejeon, 34134 Korea; 3 School of Life Sciences and Systems Biology Research Center, Gwangju Institute of Science and Technology (GIST), Gwangju, 61005 Korea; 4 Disease Target Structure Research Center, Korea Research Institute of Bioscience and Biotechnology (KRIBB), Daejeon, 34141 Korea; 5 Department of Biological Sciences, Korea Advanced Institute of Science and Technology (KAIST), Daejeon, 34141 Korea; 6 Department of Functional Genomics, University of Science and Technology (UST), Daejeon, 34141 Korea; 7 Department of Cardiology, Chonnam National University Hospital, Gwangju, Korea; 8 Department of Biochemistry, College of Life Science and Biotechnology, Yonsei University, Seoul, 03722 Korea

**Keywords:** Protein-protein interaction networks, Necroptosis, Heart failure

## Abstract

Ischaemic heart disease (IHD) is the leading cause of death worldwide. Although myocardial cell death plays a significant role in myocardial infarction (MI), its underlying mechanism remains to be elucidated. To understand the progression of MI and identify potential therapeutic targets, we performed tandem mass tag (TMT)-based quantitative proteomic analysis using an MI mouse model. Gene ontology (GO) analysis and gene set enrichment analysis (GSEA) revealed that the glutathione metabolic pathway and reactive oxygen species (ROS) pathway were significantly downregulated during MI. In particular, glutathione peroxidase 4 (GPX4), which protects cells from ferroptosis (an iron-dependent programme of regulated necrosis), was downregulated in the early and middle stages of MI. RNA-seq and qRT-PCR analyses suggested that GPX4 downregulation occurred at the transcriptional level. Depletion or inhibition of GPX4 using specific siRNA or the chemical inhibitor RSL3, respectively, resulted in the accumulation of lipid peroxide, leading to cell death by ferroptosis in H9c2 cardiomyoblasts. Although neonatal rat ventricular myocytes (NRVMs) were less sensitive to GPX4 inhibition than H9c2 cells, NRVMs rapidly underwent ferroptosis in response to GPX4 inhibition under cysteine deprivation. Our study suggests that downregulation of GPX4 during MI contributes to ferroptotic cell death in cardiomyocytes upon metabolic stress such as cysteine deprivation.

## Introduction

Heart disease is the leading cause of death worldwide, and ischaemic heart disease (IHD), also known as coronary artery disease (CAD), is the most common type of heart disease^1^. Cardiac ischaemia can induce the death of cardiomyocytes, and the damaged heart tissues are immediately replaced with fibrotic scar tissue^2^. The scar tissue consists of proliferating fibroblasts, which cannot compensate for contraction function. Therefore, the loss of cardiomyocytes eventually leads to heart failure. Various cell death pathways were suggested to contribute to the loss of cardiomyocytes during MI^3,4^. Extrinsic apoptotic signals through death receptor induce the disruption of cardiac homeostasis^5^. In addition, intrinsic apoptotic pathways lead to the disruption of mitochondrial integrity by activating proapoptotic factors, thereby contributing to the death of cardiomyocytes^6^. Opening of the mitochondrial permeability transition pore (mPTP) by the elevated Ca^2+^ levels in mitochondria is associated with necrotic cell death resulting in cardiac dysfunction^7^. In addition, recent studies reported that necroptosis, which is activated by receptor interacting protein kinases (RIPKs), represents a promising target for cardiovascular diseases^8–10^. Necrostatin-1 (Nec-1), a RIPK1 inhibitor, exhibited cardioprotective effects in various cardiovascular disease models^10–14^.

Oxidative stress is also known to mediate cardiac tissue damage by inducing cardiomyocyte death^15,16^. Reduced expression or activity of several antioxidant enzymes, including superoxide dismutase (SOD), glutathione peroxidase 1 (GPx1) and catalase (CAT), during MI has been reported to be associated with an increase in reactive oxygen species (ROS)^17^. Upon MI, the levels of glutathione (GSH), a key cellular antioxidant, were decreased in cardiomyocytes and heart tissues due to deregulated glucose metabolism and growth factor signalling, contributing to cardiomyocyte death^18,19^. Restoration of GSH levels by exogenous GSH supplementation enhanced myocardial resistance to ischaemia-reperfusion^20^. Furthermore, several antioxidants relieved the symptoms of MI in clinical studies, suggesting that excessive ROS contributes to the progression of MI^16^.

Ferroptosis is a lipid ROS-induced cell death programme that is dependent on intracellular iron and exhibits features different from those of other types of cell death^21,22^. Among the GPx family members, GPX4 is known for specifically catalysing the reduction of lipid peroxides, thereby removing lipid ROS^23^. GPX4 depletion in cells and mice results in the accumulation of lipid peroxides and lipid ROS in a 12/15-lipoxygenase (12/15-LOX)-dependent manner, leading to cell death^21,22^. The reduction of lipid peroxides by GPX4 requires the oxidation of GSH, which acts as an electron donor^23^. Since GSH is mainly synthesized from cysteine, inhibition of cystine/glutamate transporter (SLC7A11/xCT) or deprivation of cysteine results in depletion of GSH, thereby inducing ferroptosis^24–26^. In addition, an importance of the transsulfuration pathway in terms of the regulation of intracellular GSH levels and ferroptosis in certain cell types was reported^27,28^. Biochemical analysis suggests that long-chain polyunsaturated fatty acids (PUFAs), such as adrenic and arachidonic acid, anchored in several phospholipids, such as phosphatidylethanolamine, are the primary targets of lipid peroxidation^29–31^. Specific inhibitors of ferroptosis, such as ferrostatin-1 (Fer-1) and liproxstatin-1 (Lip-1), were identified, and these inhibitors act as radical-trapping antioxidants (RTAs) specifically removing radicals in phospholipids, thereby suggesting that they are much more potent than general ROS scavengers in terms of protecting cells from ferroptosis^21,32,33^.

To understand the progression of MI, we recently reported mRNA and miRNA changes during MI at different stages. Here, we reported proteomic alterations that occurred in the mouse model of MI and identified that GPX4 is a significantly downregulated protein during MI, contributing to the ferroptosis of cardiomyocytes.

### Proteome analysis reveals that GPX4 protein levels decrease during myocardial infarction

**Fig. 1:**
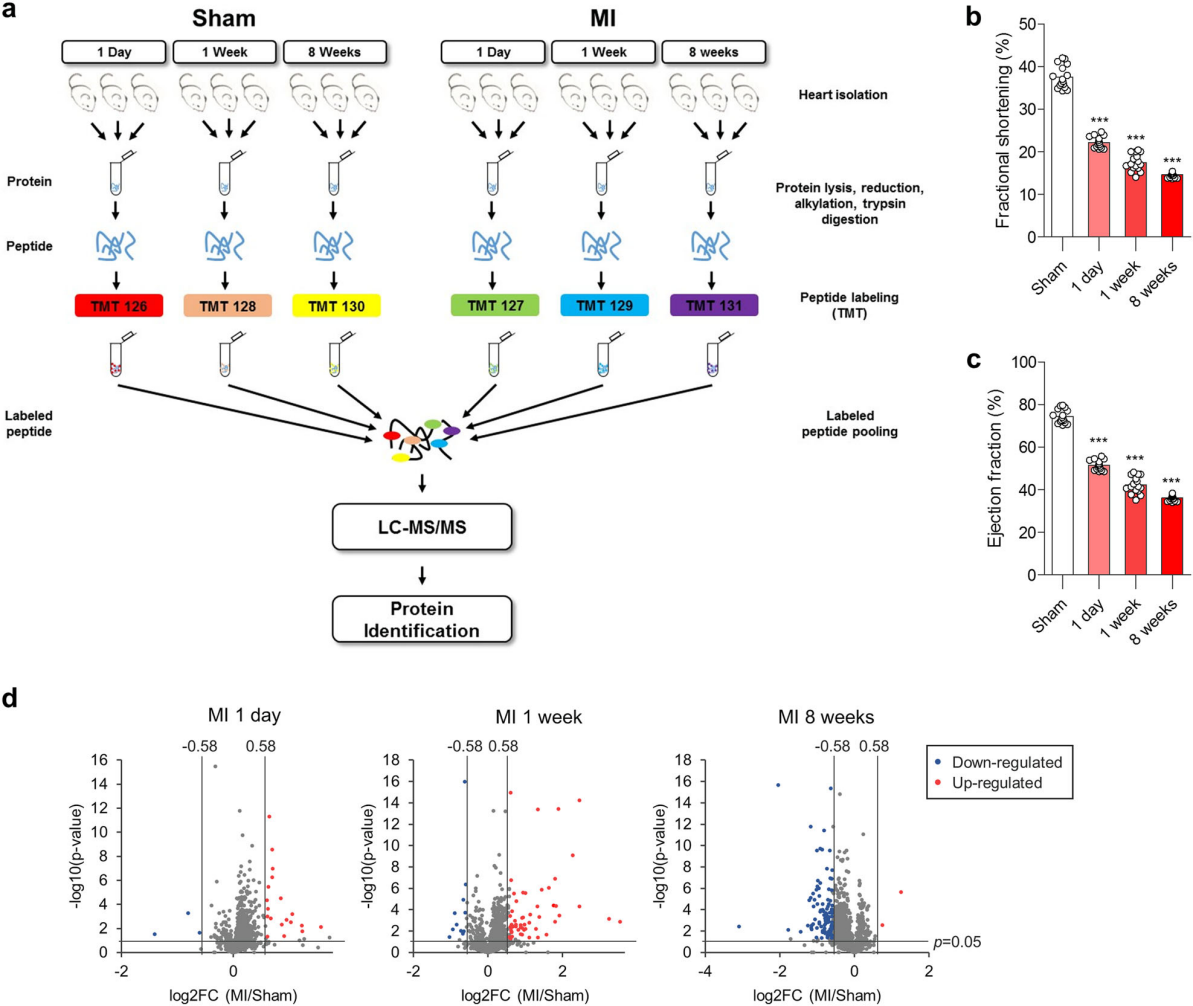
The protein and mRNA levels of GPX4 are downregulated during MI. **a** Schematic overview of tandem mass tag (TMT)-based quantitative proteomics analysis of MI mouse samples. Proteins were pooled from isolated heart tissue from mice at 1 day, 1 week, and 8 weeks of MI and from sham mice. Samples were reduced, alkylated and digested with trypsin. Samples labelled with TMT sixplex were analysed by LC-MS/MS. **b**, **c** Validation of the mouse model of MI by assessing cardiac function in terms of the fractional shortening (FS) (**b**) and LV ejection fraction (EF) (**c**) at each stage. The data are the mean ± s.d.; *n* = 16, with ***P < 0.001 compared with sham with a two-sided Student’s *t*-test. **d** Volcano plot showing proteins detected by LC-MS/MS at 1 day, 1 week, and 8 weeks post MI. Significantly upregulated or downregulated proteins (*p*-value < 0.05, |fold change| > 1.5) are marked in red or blue, respectively

To understand the molecular mechanism underlying the progression of MI, we recently generated a mouse model of MI by left anterior descending (LAD) ligation at three different stages (1 day: early; 1 week: middle; 8 weeks: late)^34^ and confirmed the hearts to be hypertrophic by the reduced left ventricular (LV) fractional shortening (FS) and ejection fraction (EF) (Fig. 1a–c). Via the echocardiography on heart tissue showing relatively equivalent damage, we analysed the changes in mRNA and miRNA levels^34^. In addition to transcriptomic analysis, we performed quantitative proteomic analysis using the same MI samples to identify key proteins involved in MI progression. Heart tissues at each MI stage were pooled. Then, proteins were isolated, labelled with TMTs, and analysed using LC-MS/MS (Fig. 1a). As a result, we identified a number of proteins and summarized significantly upregulated or downregulated proteins (adjusted *p*-value < 0.05, |fold change| > 1.5) in mice at each stage of MI compared with those in sham mice (Supplementary Tables 1–4). A volcano plot shows that the overall tendency of protein levels increased during the early and middle stages of MI, while many significantly downregulated proteins were detected at the late stage of MI (Fig. 1d).

**Fig. 2:**
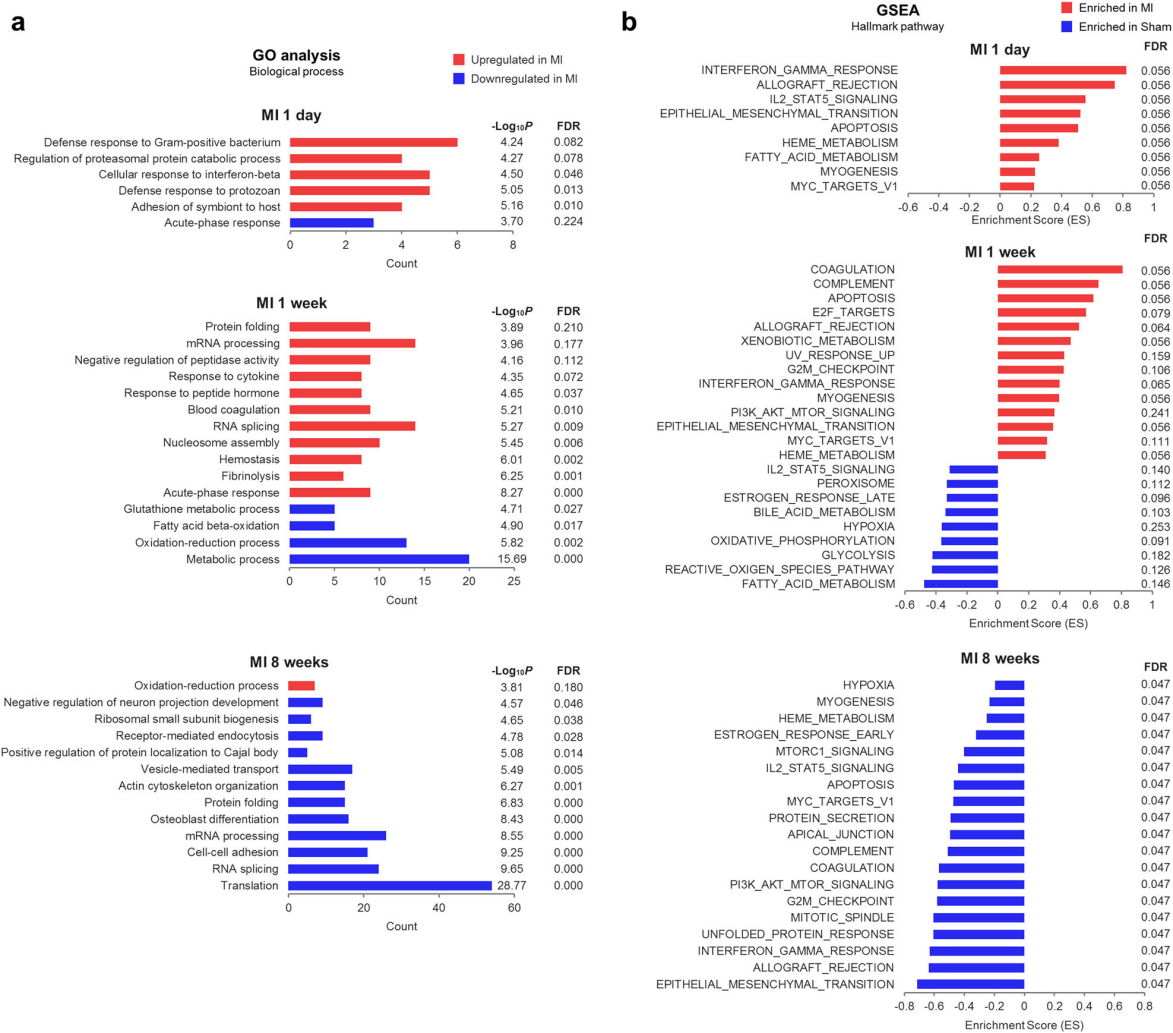
Gene ontology (GO) analysis and gene set enrichment analysis (GSEA) of proteome data from MI samples. **a** GO analysis of the differentially expressed proteins (DEPs) (|fold change| > 1.25) at each stage of MI using the DAVID database (https://david.ncifcrf.gov/). **b** GSEA of the normalized expression of all proteins at each stage of MI using the GSEA hallmark pathways database (http://software.broadinstitute.org/gsea/msigdb/)

To identify the key pathways altered in MI, we performed gene ontology (GO) analysis. GO analysis suggested that inflammation and immune pathways were strongly activated at the early and middle stages (Fig. 2a). In addition, several metabolic pathways such as the GSH, fatty acid, and oxidation-reduction pathways were downregulated at the early stage (Fig. 2a). In contrast, the most prominently altered processes at the late stage of MI were the inhibition of translation, RNA splicing and mRNA processing (Fig. 2a). Furthermore, many cellular pathways were deregulated, suggesting that the overall function of cardiomyocytes was collapsed (Fig. 2a). To identify the specific signalling pathways in detail, gene set enrichment analysis (GSEA) was performed. The results show that the interferon, IL-2-STAT5, and epithelial-mesenchymal transition (EMT) pathways were upregulated at the early stage of MI, while the ROS pathway, glycolysis, and fatty acid metabolism were downregulated at the middle stage (Fig. 2b). These results are correlated with our previous GSEA from transcriptome data, confirming the significant role of these pathways during the progression of MI^34^.

**Fig. 3:**
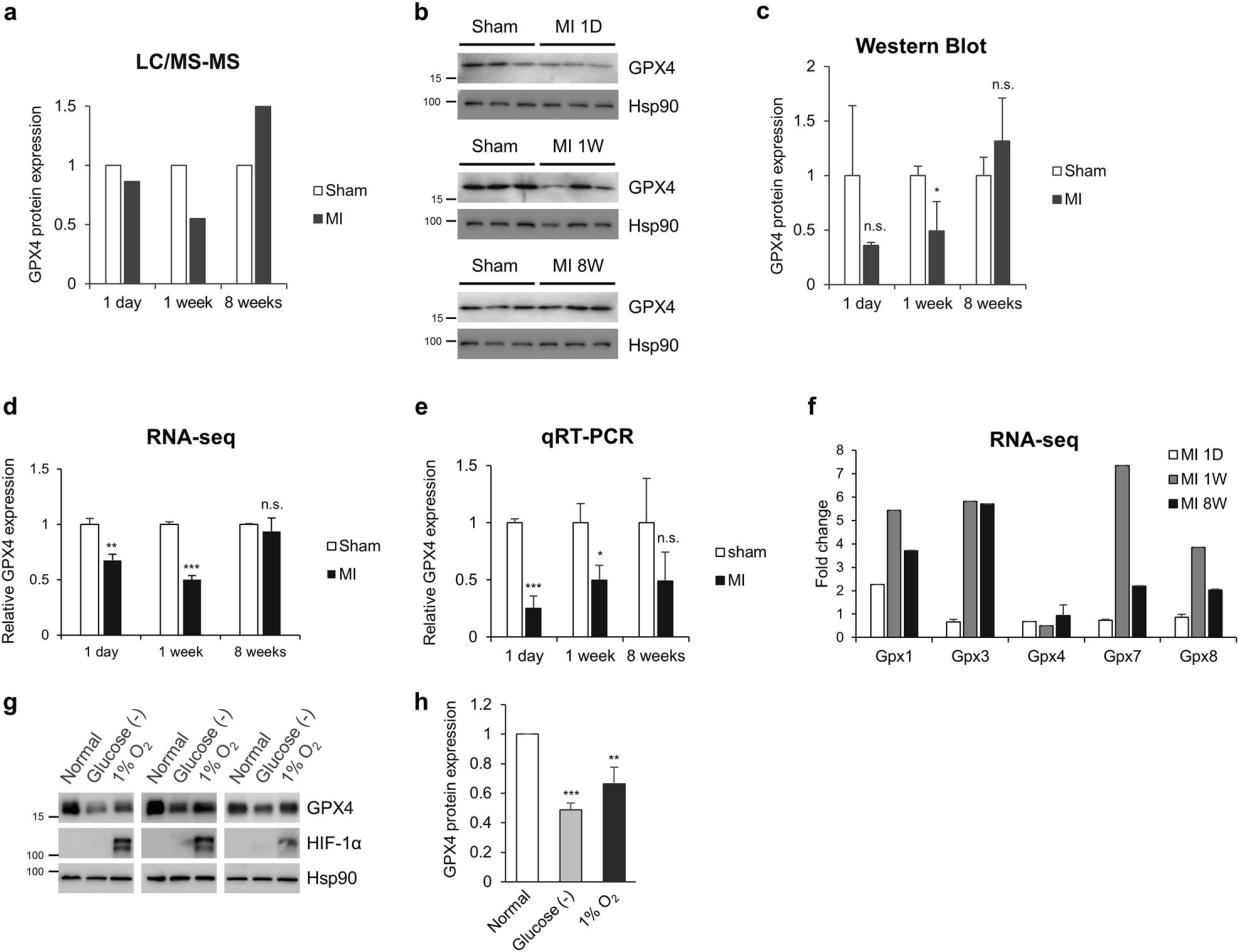
GPX4 is downregulated during MI progression. **a** Expression of GPX4 protein detected by LC-MS/MS in samples from mice at the different stages of MI and in sham mouse samples. **b**, **c** Validation of GPX4 protein expression by western blot analysis. Left ventricular (LV) tissues of MI and sham mice were lysed and subjected to western blot analysis using an anti-GPX4 antibody (**b**). The amount of GPX4 protein was quantified by ImageJ and is shown in (**c**). The data are the mean ± s.d.; *n* = 3, with *P < 0.05 and n.s. = nonsignificant compared with sham with a two-sided Student’s *t*-test. **d** Expression of GPX4 mRNA from LV tissues of MI mouse samples determined by RNA-sequencing (RNA-seq). The data are the mean ± s.d.; *n* = 3, with **P < 0.01, ***P < 0.001 and n.s. = nonsignificant compared with sham with a two-sided Student’s *t*-test. **e** Validation of GPX4 mRNA expression by qRT-PCR analysis. The data are the mean ± s.d.; *n* = 3, with *P < 0.05, ***P < 0.001 and n.s. = nonsignificant compared with sham with a two-sided Student’s *t*-test. **f** Comparison of the mRNA expression levels of the GPX family from RNA-seq data. **g**, **h** Expression of GPX4 under ischaemic conditions in vitro. H9c2 cells were incubated in glucose-deficient medium or under hypoxic conditions (1% O_2_). The amount of GPX4 protein was quantified by ImageJ and is shown in (**h**). HIF-1 was also assessed as a hypoxia marker. The data are the mean ± s.d.; *n* = 3, with **P < 0.01 and ***P < 0.001 compared with normal with a two-sided Student’s *t*-test

In particular, several proteins involved in GSH metabolism such as GPX4, glutathione S-transferase mu 2 (GSTM2), and GSTM4 were significantly downregulated in mice at the middle stage of MI (Supplementary Table 3)^35^. Among these proteins, we focused on the GPX4 protein, a key enzyme that protects cells from ferroptosis by removing lipid hydroperoxide^36^. LC-MS/MS analysis indicated that protein levels of GPX4 decreased in mice at the early and middle stages of MI and increased in mice at the late stage (Fig. 3a). In agreement with the mass spectrometry results, western blot analysis showed that GPX protein levels were indeed downregulated at the early and middle stages of MI samples, while they were slightly increased at the late stage (Fig. 3b, c). We next asked whether the altered protein levels of GPX4 were due to reduced mRNA expression. RNA-seq results showed the lower mRNA levels of GPX4 in mice at the early and middle stages of MI compared with those in control mice (Fig. 3d)^34^. When analysing by qRT-PCR analysis, we found that GPX4 mRNA levels were indeed downregulated during MI (Fig. 3e). However, the mRNA expression levels of other GPx family members were increased (Fig. 3f), possibly due to a compensatory antioxidant response against ROS, suggesting that GPX4 is a specifically downregulated protein among GPx family members during MI. To investigate whether GPX4 protein levels are directly controlled by ischaemic stress, H9c2 cardiomyocytes were incubated in glucose-deprived medium or under hypoxic conditions (1% O_2_). GPX4 protein levels were decreased following exposure to glucose deficiency or hypoxia, suggesting that ischaemic stress might directly induce the downregulation of GPX4 protein levels (Fig. 3g, h). Since many cellular pathways including inflammatory and metabolic pathways are altered during myocardial infarction in vivo (Fig. 2), we could not exclude the possible contribution of the cardiac microenvironment to GPX4 protein levels and ferroptosis sensitivity^37,38^. Collectively, our proteome analysis revealed that GPX4 protein levels decreased during the early and middle stages of MI and that downregulation of GPX4 might contribute to ferroptotic cell death of cardiomyocytes during MI.

### Inhibition of GPX4 induces myocardial ferroptosis

**Fig. 4:**
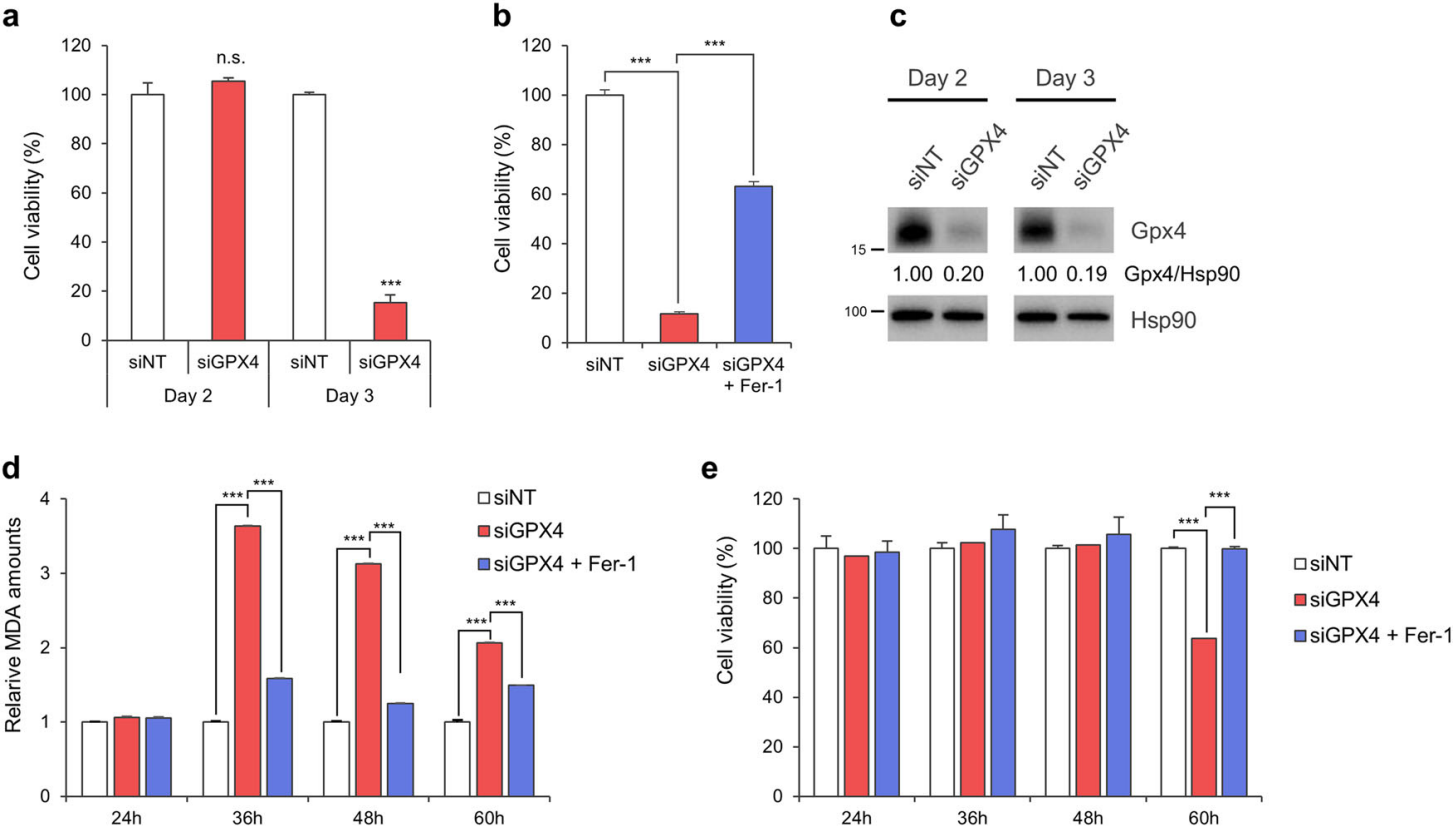
Downregulation of GPX4 induces ferroptosis by increasing lipid ROS levels in H9c2 cells. **a** Viability of H9c2 cells depleted of GPX4. H9c2 cells were transfected with 20 nM non-targeting siRNA pool (siNT pool) or GPX4-targeting siRNA (siGPX4 pool) for 2 and 3 days. Cell viability was measured using CellTiter-Glo. The data are the mean ± s.d.; *n* = 3, with ***P < 0.001 and n.s. = nonsignificant compared with siNT with a two-sided Student’s *t*-test. **b** Inhibition of GPX4 knockdown-induced ferroptosis by Fer-1. H9c2 cells were transfected with siGPX4 for 72 h. Then, 1 μM Fer-1 was added 24 h after siRNA transfection. Cell viability was measured using CellTiter-Glo. The data are the mean ± s.d., *n* = 3, with ***P < 0.001 by a two-sided Student’s *t*-test. **c** Western blot analysis of H9c2 cells transfected with GPX4 siRNA. H9c2 cells were transfected with the indicated siRNAs for 2 and 3 days. Cells were then lysed and subjected to western blot analysis using an anti-GPX4 antibody. The relative amounts of GPX4 were calculated and are shown after normalizing to Hsp90. **d**, **e** Comparison between lipid peroxidation and cell viability upon GPX4 depletion. H9c2 cells were transfected with siGPX4 for the indicated periods in the absence or presence of 1 μM Fer-1. Lipid peroxidation was determined by measuring the amount of malondialdehyde (MDA) using a lipid peroxidation assay kit, and cell viability was measured using CellTiter-Glo (**e**). The data are the mean ± s.d., *n* = 3, with ***P < 0.001 with a two-sided Student’s *t*-test

To directly assess the involvement of GPX4 in myocardial cell death, we used a GPX4 siRNA pool consisting of four independent siRNAs. While cells transfected with GPX4 siRNA for 2 days were viable, cells depleted for GPX4 for 3 days completely died (Fig. 4a). Cell death induced by GPX4 depletion was prevented by Fer-1, suggesting that GPX4 depletion induced ferroptosis in H9c2 cells (Fig. 4b). Western blot analysis showed that GPX4 was efficiently depleted in cells transfected with GPX4 siRNA for 2 days, suggesting that an additional period is required to undergo ferroptosis following GPX4 depletion (Fig. 4a, c). Notably, the amount of malondialdehyde (MDA), one of the most prevalent by-products of lipid peroxidation, was markedly increased at 36 h after GPX4 siRNA transfection and gradually decreased over time (Fig. 4d). In addition, the production of lipid peroxides upon GPX4 knockdown was inhibited by Fer-1 (Fig. 4d). However, the cells did not die until 48 h of GPX4 knockdown and underwent ferroptotic cell death at 60 h, which was prevented by Fer-1 (Fig. 4e). These data suggest that GPX4 deficiency induced the accumulation of lipid ROS at the early phase and then caused ferroptosis after a period of time.

**Fig. 5:**
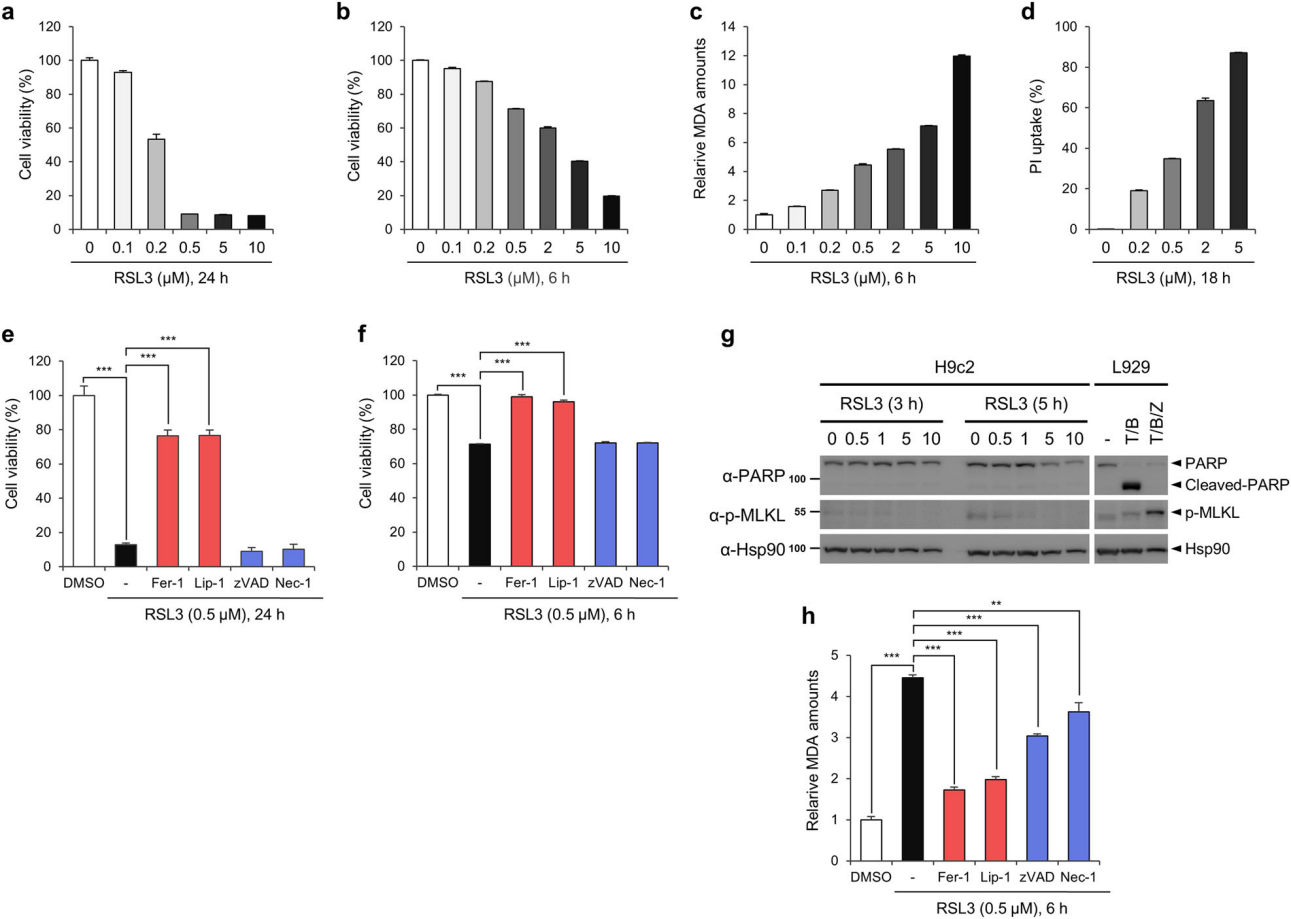
H9c2 cells are sensitive to RSL3-induced ferroptosis. **a** Viability of H9c2 cells treated with RSL3. H9c2 cells were treated with increasing concentrations of RSL3 for 6 h. Cell viability was measured using CellTiter-Glo. **b**, **c** Cell viability and lipid ROS analysis of H9c2 cells treated with RSL3. H9c2 cells treated with RSL3 for 6 h were subjected to a CellTiter-Glo assay (**b**) and an MDA assay (**c**). **d** Cell death determined by propidium iodide (PI) uptake. H9c2 cells were treated for 18 h and then treated with 10 μg/mL PI for 30 min prior to harvest. The number of PI-positive cells was determined by flow cytometry. **e**, **f** Inhibition of RSL3-induced cell death by ferroptosis inhibitors. H9c2 cells were treated with 0.5 μM RLS3 in the presence of ferroptosis inhibitors (ferrostatin-1, Fer-1; liproxstatin-1, Lip-1), an apoptosis inhibitor (zVAD-fmk, zVAD), or a necroptosis inhibitor (necrostatin-1, Nec-1) for 24 h and 6 h, and cell viability was measured. The data are the mean ± s.d.; *n* = 3, with ***P < 0.001 with a two-sided Student’s *t*-test. **g** Western blot analysis showing that neither apoptosis (PARP cleavage) nor necroptosis (phospho-MLKL) was activated by RSL3 in H9c2 cells. L929 cells treated with rat TNF/birinapant (T/B) or TNF/birinapant/zVAD-fmk (T/B/Z) were used as positive controls for apoptosis and necroptosis, respectively. **h** Prevention of RSL3-induced lipid ROS accumulation by ferroptosis inhibitors. Cells were treated with RSL3 and inhibitors as described in (**f**) for 6 h, and lipid peroxidation was assessed using a lipid peroxidation assay. The data are the mean ± s.d., *n* = 3, with **P < 0.01, ***P < 0.001 with a two-sided Student’s *t*-test

To further analyse the relationship between lipid ROS and ferroptosis, cells were treated with increasing concentrations of RSL3, a GPX4 inhibitor. Treatment of H9c2 cells with 0.2 μM RSL3 for 24 h decreased cell viability by ~50%, while almost all cells treated with 0.5 μM RSL3 died (Fig. 5a). Since lipid peroxidation rapidly accumulates and disappears before the cells die, we determined MDA levels in cells treated with RSL3 for 6 h. Short-term treatment of cells with RSL3 also induced cell death in a concentration-dependent manner (Fig. 5b). As the amount of lipid ROS was gradually increased by RSL3 treatment, cell death increased, implying that certain levels of lipid ROS might be required for the execution of ferroptosis (Fig. 5b, c). RSL3 also induced cell death with a necrosis-like morphology and increased the propidium iodide (PI)-positive cell population, suggesting that RSL3-induced cell death is necrotic (Fig. 5d and Supplementary Fig. 1a). To address the possible involvement of other cell death pathways, we employed several cell death inhibitors. When cells were treated with RSL3 in the presence of ferroptosis inhibitor Fer-1 or Lip-1, cell death was prevented (Fig. 5e, f). However, RSL3-induced cell death was not inhibited by zVAD or Nec-1, and no PARP cleavage or MLKL phosphorylation was observed in RSL3-treated cells, suggesting that apoptosis and necroptosis are not involved in this pathway (Fig. 5e–g). Consistent with these results, RSL3-induced lipid ROS levels were abrogated by Fer-1 and Lip-1 but not by zVAD or Nec-1, indicating that lipid ROS generated by RSL3 treatment resulted in ferroptosis in H9c2 cells (Fig. 5h).

### Depletion of intracellular GSH upon cysteine starvation induces ferroptosis

**Fig. 6:**
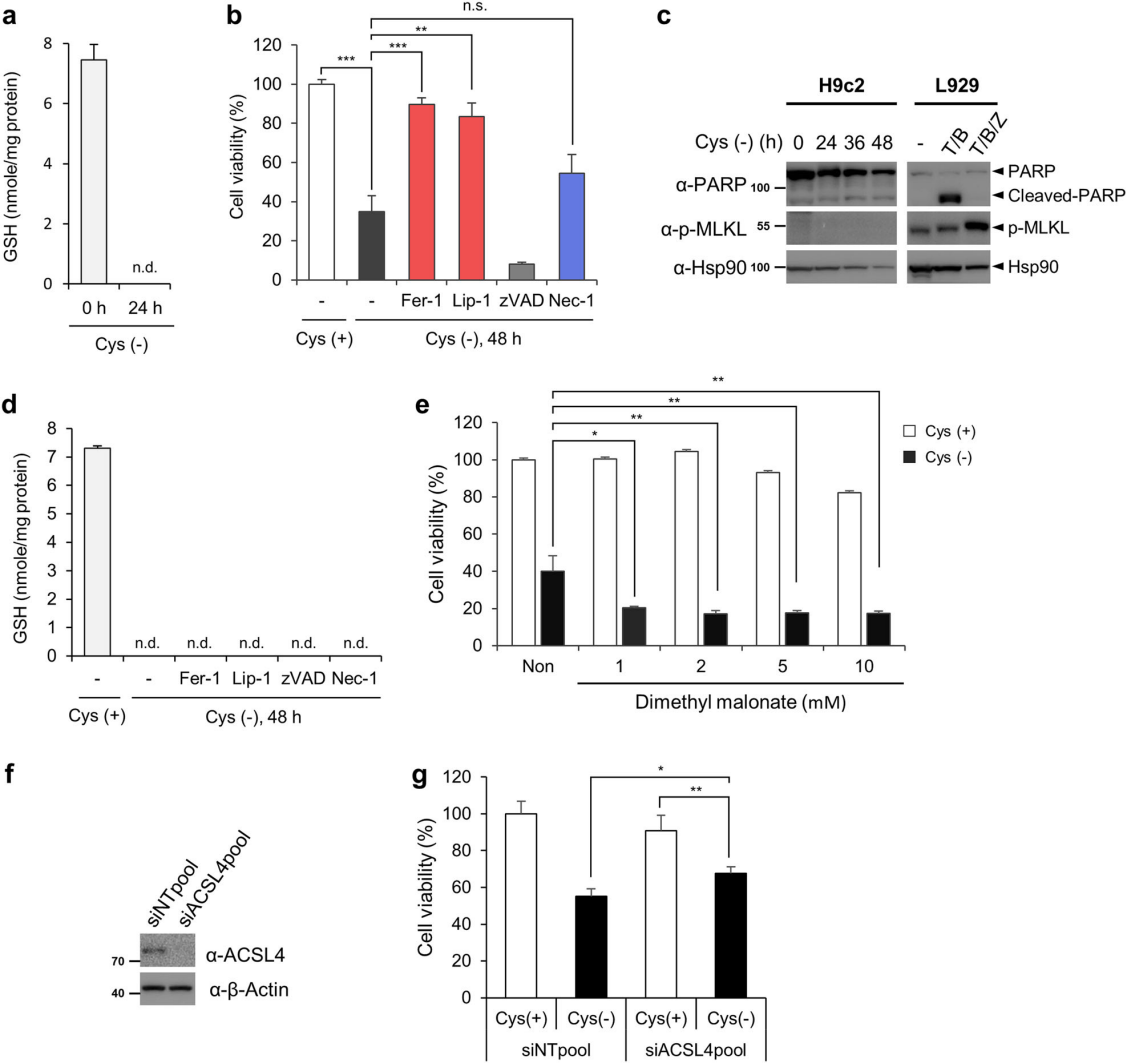
Cysteine deprivation induces glutathione depletion and ferroptosis in H9c2 cells. **a** Determination of glutathione levels upon cysteine deprivation. H9c2 cells were incubated in cysteine- and methionine-deficient medium for 24 h. Glutathione levels were measured using a glutathione assay kit according to the manufacturer’s instructions and normalized to protein concentrations. n.d. = not determined. **b** Effect of cysteine deprivation on ferroptosis in H9c2 cells. H9c2 cells were incubated in cysteine-depleted DMEM for 48 h in the absence or presence of inhibitors, as described in the legend of Fig. 5e. Cell viability was measured using CellTiter-Glo. The data are the mean ± s.d., *n* = 3, with **P < 0.01, ***P < 0.001 and n.s. = nonsignificant with a two-sided Student’s *t*-test. **c** Western blot analysis of H9c2 cells cultured in cysteine-deficient medium. Cells were incubated in cysteine-deficient medium for the indicated periods, and western blotting was carried out as described in the legend of Fig. 5g. **d** Determination of glutathione levels upon cysteine deprivation in the presence of inhibitors. H9c2 cells were treated as in (**b**) for 40 h, and glutathione levels were determined. **e** H9c2 cells were incubated in cysteine-depleted medium in the absence and presence of 10 mM DMM. Cell viability was measured with CellTiter-Glo. The data are the mean ± s.d., *n* = 3, with *P < 0.05 and **P < 0.01 with a two-sided Student’s *t*-test. **f** H9c2 cells transfected with ACSL4 siRNA pool for 48 h and western blot analysis was carried out using anti-ACSL4 antibody. **g** H9c2 cells transfected with ACSL4 siRNA for 36 h were incubated in cysteine-depleted medium for an additional 48 h. The data are the mean ± s.d., *n* = 3, with *P < 0.05 and **P < 0.01 with a two-sided Student’s *t*-test

In physiologic conditions, MI involves ischaemic stress resulting in the depletion of metabolites, including GSH^18,19^. Because the impact of cysteine depletion on cardiomyocyte death has not been directly assessed, we tested the effect of cysteine deficiency on ferroptosis in cardiomyocytes. As shown in the previous reports^39,40^, we also observed a nearly complete depletion of intracellular GSH in cells cultured in cysteine-deficient medium for 24 h (Fig. 6a). Similar to GPX4 depletion, cells did not die at 24 h cysteine deprivation condition, denoting that GSH depletion may require an additional period of time to induce ferroptosis. Cysteine depletion for 48 h reduced H9c2 cell viability, which was completely rescued by the presence of Fer-1 or Lip but not by zVAD (Fig. 6b). Furthermore, cysteine deprivation did not induce PARP cleavage, indicating that cysteine deficiency-induced cell death is ferroptotic but not apoptotic. However, interestingly, although Nec-1, a RIPK1 inhibitor, did not affect RSL3-induced cell death, it partially prevented cell death induced by cysteine deficiency (Fig. 6b). Since no MLKL phosphorylation was observed, these results suggest that RIPK1-dependent and MLKL-independent signalling might be involved in cysteine deficiency-induced ferroptosis as previously suggested in breast cancer cells (Fig. 6b, c)^39^. Similarly, erastin, which inhibits the cystine-glutamate antiporter system *X*_c_^−^, was previously shown to induce Fer-1- and Nec-1-sensitive cell death in certain cell types^33,41^. Since Fer-1 and Lip are known to be radical-trapping agents, the intracellular GSH level was unchanged in the presence of Fer-1 or Lip (Fig. 6b, d).

Recently, succinate dehydrogenase was shown to produce ROS during ischaemia-reperfusion injury in the heart and brain^42^. To address whether succinate dehydrogenase is required for ferroptosis in cardiomyocytes, we employed dimethyl malonate (DMM), an inhibitor of succinate dehydrogenase^42,43^. However, neither cysteine deprivation- nor RSL3-mediated ferroptosis was affected by DMM, suggesting that cardiomyocytes might use a different ROS source than mitochondrial complex II (Fig. 6e and Supplementary Fig. 2a). Long-chain acyl-CoA synthetase 4 (ACSL4), which mediates the generation of PUFA-phosphatidylethanolamine, is an essential enzyme in most ferroptosis pathways^29,30^. While ACSL4 depletion slightly ameliorated cysteine deprivation-induced ferroptosis, it showed little effect on RSL3-induced ferroptosis (Fig. 6f, g and Supplementary Fig. 2b). Since ACSL4 is dispensable for p53-induced ferroptosis, other ACSL family members might also be involved in ferroptosis in cardiomyocytes^44^. These data imply that cardiomyocytes undergo ferroptosis upon cysteine deprivation or GPX4 inhibition, but the underlying mechanisms might differ from those in other type of cells.

### Inhibition of GPX4 sensitizes primary NRVM cells to ferroptosis upon cysteine starvation

**Fig. 7:**
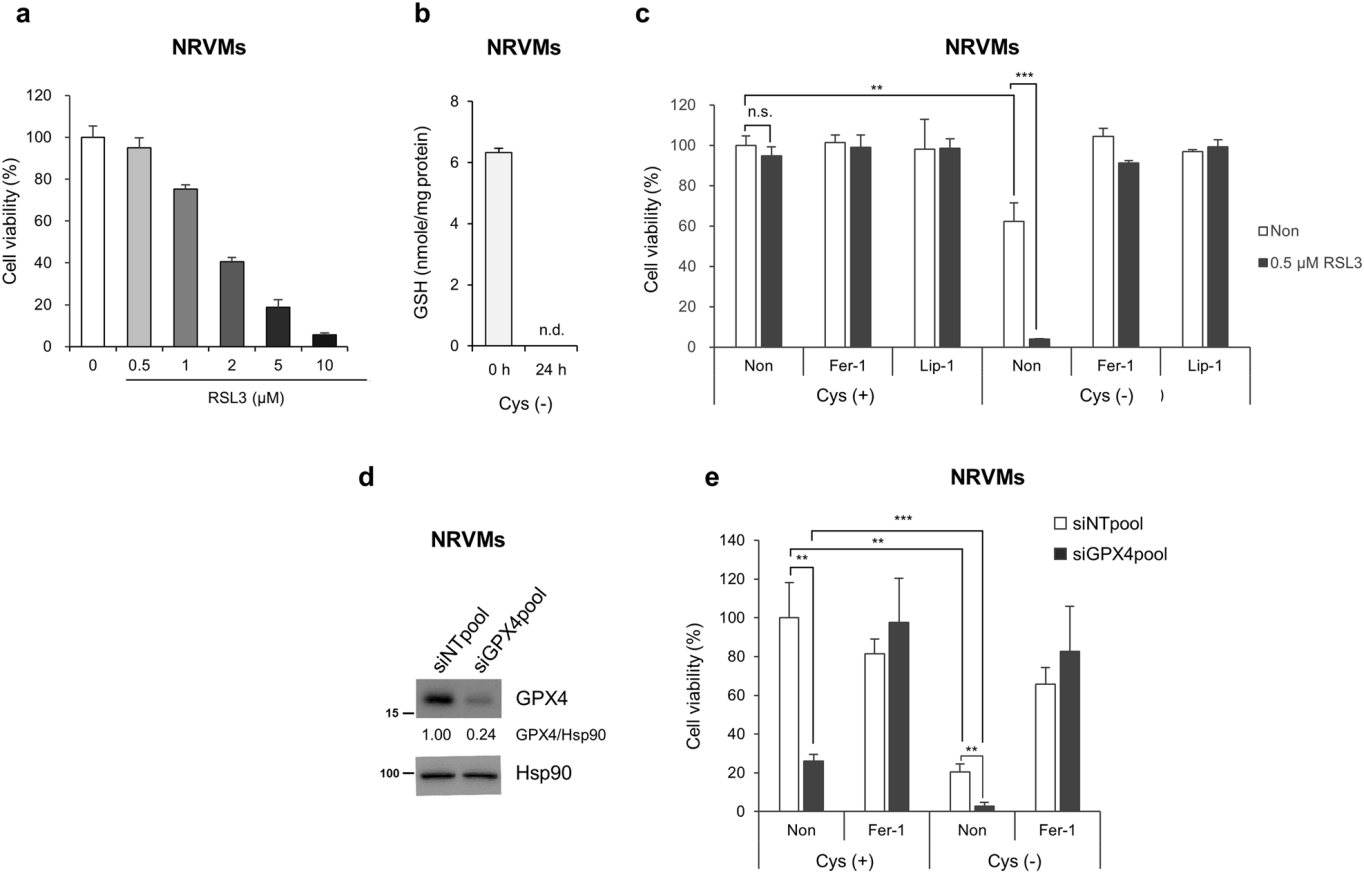
Inhibition of GPX4 sensitizes NRVMs to ferroptosis in the absence of cysteine. **a** Cell viability of neonatal rat ventricular myocytes (NRVMs) treated with RSL3. NRVMs were treated with different concentrations of RSL3 for 24 h, and cell viability was analysed as described above. **b** NRVMs were incubated in cysteine- and methionine-deficient medium for 24 h. Glutathione levels were measured using a glutathione assay kit according to the manufacturer’s instructions and normalized to protein concentrations. n.d. = not determined. **c** RSL3-induced ferroptosis in NRVMs cultured in cysteine-depleted medium. NRVMs were incubated with normal DMEM or cysteine-depleted DMEM. After 24 h, cells were treated with 0.5 μM RSL3 for an additional 24 h, and cell viability was analysed as described above. The data are the mean ± s.d., *n* = 3, with **P < 0.01, ***P < 0.001 and n.s. = nonsignificant with a two-sided Student’s *t*-test. **d** siRNA-mediated knockdown of GPX4 in NRVMs. NRVMs were transfected with 20 nM GPX4 siRNA for 48 h and subjected to western blot analysis. **e** Effect of a combination of GPX4 knockdown and cysteine deprivation on ferroptosis in NRVMs. NRVMs were transfected with 20 nM siGPX4. After 24 h, the medium was replaced with cysteine-deficient medium with or without 1 μM Fer-1, and the cells were incubated for an additional 48 h. Cell viability was analysed as described above. The data are the mean ± s.d., *n* = 3, with **P < 0.01 and ***P < 0.001 with a two-sided Student’s *t*-test

We next tested the role of GPX4 on primary neonatal rat ventricular myocytes (NRVMs). While H9c2 cells almost completely died in the presence of 0.5 μM RSL3, NRVMs did not (Fig. 5a, and 7a). However, a high concentration of RSL3-induced cell death in NRVMs, suggesting that NRVMs are somehow less sensitive than H9c2 cells to ferroptosis (Fig. 7a). Interestingly, undifferentiated C2C12 cells, a different type of myocyte, were also highly sensitive to RSL3-induced ferroptosis, while differentiated C2C12 cells were relatively resistant to ferroptosis (Supplementary Fig. 3). Since myocyte differentiation induces metabolic and signalling reprogramming^45^, these changes might induce the signalling system to acquire resistance to ferroptosis. Thus, although cysteine deficiency induced GSH depletion and ferroptosis in NRVMs, its effect on ferroptosis in NRVMs was weaker than that in H9c2 cells (Figs. 6a, b, 7b, c). Notably, although a low dose of RSL3 (0.5 μM) was insufficient to induce ferroptosis in NRVMs, it induced massive NRVM death by ferroptosis when cysteine was deprived (Fig. 7c). Furthermore, we found that GPX4-depleted cells underwent some degree of ferroptosis under normal conditions, which was further enhanced in the absence of cysteine (Fig. 7d, e). Taken together, our data suggest that GPX4 is downregulated during MI, which sensitizes cardiomyocytes to ferroptosis when GSH levels are decreased.

### NRF2 and EMT pathways are significantly altered during MI

**Fig. 8:**
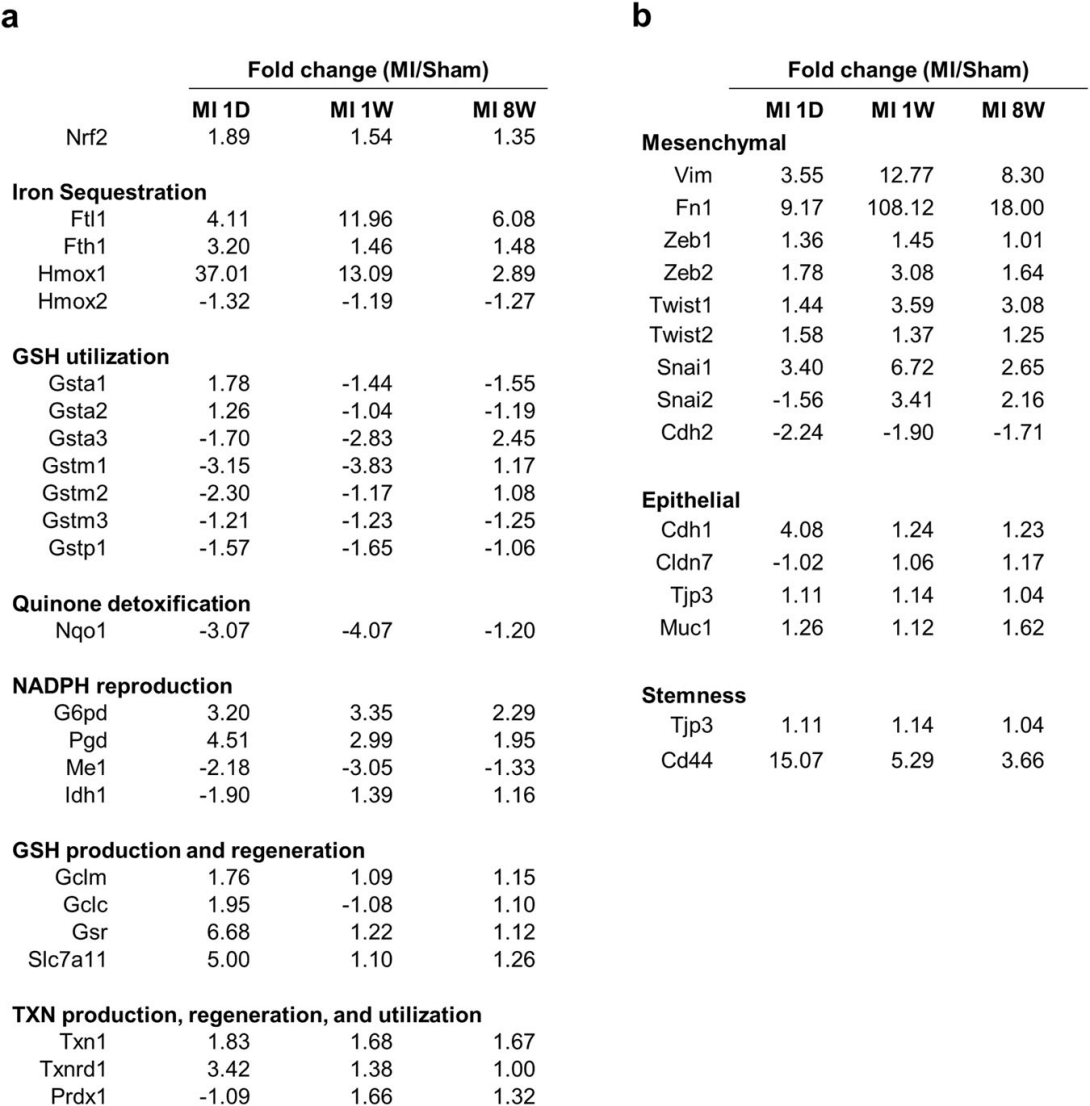
mRNA expression levels from RNA-seq data at each stage of MI. **a** mRNA expression of NRF2 and its target genes. **b** mRNA expression of EMT-related genes

During MI progression, various signalling pathways are altered and may modulate sensitivity to ferroptosis. GSEA analyses from proteome and transcriptome data identified that the ROS pathway is significantly deregulated at the middle stage of MI (Fig. 2)^34^. NRF2 is the master transcription factor controlling antioxidant responses and protects cells from lethal ROS stress^46,47^. In addition, NRF2 plays a key role in suppressing ferroptosis in various types of cells^48–50^. Interestingly, the mRNA levels of NRF2 and several target genes increased following MI (Fig. 8a). In particular, the mRNA levels of ferritin light chain 1 (Ftl1), ferritin heavy chain 1 (Fth1), and haem oxygenase 1 (Hmox1) were strongly increased in MI (Fig. 8a). In addition, several genes involved in NADPH, GSH, and TXN metabolism were upregulated in MI (Fig. 8a). Since MI involves ROS induction, activation of NRF2 antioxidant programme might be a feedback response to protect cardiac tissues from severe damage to some extent. In contrast, several antioxidant targets of NRF2 such as Gstm, Nqo1 and Me1 are downregulated during MI. Although NRF2 is a central regulator of the antioxidant programme, antioxidant genes seem to be differentially regulated, possibly by other transcription factors or cofactors. In addition, it is unclear how NRF2 suppresses ferroptosis. Quinone oxidoreductase-1, Hmox1 and Fth1 have been suggested to be the major targets of NRF2 that inhibit ferroptosis^50^. However, HO-1 was recently shown to contribute to ferroptosis by producing excessive amounts of iron in the labile Fe(II) pool, a finding that argues for a specific role of the NRF2 target in ferroptosis regulation^51,52^. Our data suggest that Hmox1 activity is largely enhanced during the early and middle stages of MI, suggesting that excessive iron could sensitize cardiac cells to ferroptosis. Since antioxidant gene induction is not consistent, the outcome of oxidative stress remains unclear.

Recently, EMT was shown to be closely linked to ferroptosis sensitivity, possibly via ZEB1-mediated lipogenic reprogamming^53^. Our proteome and transcriptome analysis showed an increase in the EMT pathway at the early stage of MI (Figs. 2 and 8b)^34^. In particular, EMT-related transcription factors such as Zeb1/2, Twist1/2 and Snail1/2 were upregulated in post-MI tissue. Furthermore, vimentin (Vim) and fibronectin-1 (FN-1) were highly expressed throughout MI progression for tissue repair as previously reported (Fig. 8b)^54–56^. Interestingly, the mesenchymal marker Cdh2 was downregulated, while the epithelial marker Cdh1 was increased during MI, suggesting that EMT-related signalling rather than the EMT process seems to be associated with myocardial infarction (Fig. 8b). Thus, activation of EMT signalling induces an intracellular environment favouring ferroptotic death of myocardial cells during MI.

## Discussion

Emerging studies have revealed that ferroptosis is implicated in several diseases, such as neurodegenerative diseases, ischaemia/reperfusion injury of the kidney, and cancer^33,57^. Several recent studies provide evidence that ferroptosis also contributes to cardiac diseases. In a mouse model of cardiac ischaemia-reperfusion injury, the iron chelator desferrioxamine (DFO) and the glutaminolysis inhibitor Compound 968, both of which inhibit ferroptosis, reduced myocardial infarct size and improved cardiac function^26^. Fer-1, a ferroptosis inhibitor, was shown to suppress cardiomyopathy in a doxorubicin-induced mouse model^58^. In addition, *GPX4*^+/−^ mice fed a high-fat, high-sucrose diet showed an increase in lipid peroxides with enhanced fibrosis and cardiomyopathy^59^. Since inhibition of system *X*_c_^−^ by erastin or an excessive amount of Fe(III)-citrate can induce ferroptosis in cardiomyocytes^60,61^, it is assumed that ischaemic injury in cardiac tissues involves depletion of metabolites such as GSH, leading to myocardial ferroptosis, which eventually induces heart failure. In addition to environmental changes, we proposed here that MI induces the downregulation of GPX4, which could sensitize cardiac cells to ferroptosis in low GSH conditions.

Accumulation of lipid ROS is the key feature of ferroptosis, but the source of ROS is currently unclear. Although depletion of mitochondria or inhibition of mitochondrial complexes delayed the rate of cysteine deprivation-induced ferroptosis, it did not completely abrogate ferroptosis^62,63^. In addition to mitochondria, the ER and lysosomes are also known to provide lipid ROS^64^. Although both compartments contribute to lipid ROS production and ferroptosis, neither compartment is essential for the execution of ferroptosis^25,64,65^. Our data suggest that in cardiomyocytes, inhibition of succinate dehydrogenase, which plays a crucial role in generating ROS during ischaemia-reperfusion injury, did not prevent cysteine deprivation-induced ferroptosis (Fig. 6e). These data suggest that cardiomyocytes might utilize a different ROS source for ferroptosis. Interestingly, we found that DMM slightly enhanced both cysteine deprivation- and RSL3-induced ferroptosis in cardiomyocytes (Fig. 6e and Supplementary Fig. 2b). As several studies have suggested, inhibition of mitochondrial complex I or depletion of mitochondria can sensitize cells to RSL3-induced ferroptosis, but the underlying mechanism is unknown^62,66^. Furthermore, a study showed that inhibition of each mitochondrial complex did not affect RSL3-induced ferroptosis but greatly suppressed cysteine deprivation-induced ferroptosis^63^. These observations suggest different mechanisms or kinetics in GPX4 inhibition-induced ferroptosis and cysteine deprivation-induced ferroptosis^63^. Therefore, identifying crucial pathways in the production of myocardial infarction-associated lipid ROS is important, and further investigation is needed.

A recent study that elaborately analysed the cardiac stem cell population using single-cell mRNA sequencing and Ki67-lineage tracing found that cardiac stem cells do not exist in damaged adult hearts and that therefore cardiomyocytes cannot be regenerated^67^. Damage in cardiac tissues induces the formation of scar tissue to replace the cardiac muscle by facilitating the proliferation of cardiac fibroblasts, thereby preventing cardiac rupture^2,67^. However, cardiac tissue replaced with scar tissue has no cardiac contraction function^2^; thus, preventing cardiomyocyte loss is critical to prevent heart failure. Indeed, many attempts to inhibit fibrosis, cardiomyocyte cell death, and oxidative stress have been tried to eventually protect cardiac tissue^2^. With analysis of recent reports, we suggest that ferroptosis during MI also contributes to cardiomyocyte death and cardiac damage, in part due to a reduction in GPX4 protein. Thus, the identification of mechanisms by which MI suppresses the transcription of GPX4 might provide a novel therapeutic approach to protect cardiomyocytes from ferroptosis and to control heart failure upon MI.

## Materials and methods

### Generation of the MI mouse model

A myocardial infarction (MI) mouse model was previously described^34,68^. Briefly, C57BL/6 mice (8-week-old males) were subjected to an MI or sham operation. After anaesthetization, the proximal left anterior descending (LAD) coronary artery was ligated, and sham-operated animals underwent the same procedure without occlusion of the LAD coronary artery. Then, 1 day, 1 week and 8 weeks after MI, the mice were sacrificed, and their hearts were removed for analysis of protein and mRNA.

### Mass spectrometric analysis

LV heart tissues from three mice at each MI stage and corresponding sham mice were pooled. Tissues were lysed in 7 M urea, and the lysates were denatured, reduced, and digested with trypsin. Samples were labelled with tandem mass tag (TMT) sixplex reagents using a TMT Mass Tagging Kit (Thermo Scientific) according to the manufacturer’s instructions. Labelled samples were analysed with an EASY-nLC1000/LTQ Orbitrap Elite.

### Gene ontology (GO) analysis and gene set enrichment analysis (GSEA)

Gene ontology (GO) and Kyoto Encyclopedia of Genes (KEGG) pathway analysis was performed as follows. GO and KEGG pathway analyses of the differentially expressed proteins (DEPs) (|fold change| > 1.25) at each stage of MI were performed using DAVID 6.7 or Cytoscape with the ClueGO plug-in. The *p*-value was calculated using right-sided hypergeometric tests and the Benjamini-Hochberg correction for multiple testing.

For the identification of enriched proteome signatures, we used the gene set enrichment analysis (GSEA) tool (v3.0) from the Broad Institute at the Massachusetts Institute of Technology. GSEA was performed by comparing normalized protein expression data obtained from the three different stages of MI. We used hallmark gene sets from MSigDB to interpret the proteomic signatures during the progression of MI.

### Analysis of RNA-seq data

We previously reported RNA-seq data from mouse LV tissues of MI or sham animals (GSE114695)^34^. The expression levels of GPx family members, genes involved in the NRF pathway, and EMT-related genes were re-analysed from the RNA-seq data.

### Cell culture, siRNA transfection and cysteine deprivation

H9c2 and C2C12 cells were maintained in Dulbecco’s modified Eagle’s medium (DMEM, HyClone-Thermo Scientific, South Logan, UT, USA) supplemented with 10% foetal bovine serum (GIBCO) and 1% penicillin/streptomycin (Invitrogen, Carlsbad, CA) in 5% CO_2_ at 37 °C. Hypoxic conditions were achieved by incubating cells in an environment containing 1% O_2_, 94% N_2_, and 5% CO_2_ in a multigas incubator (Sanyo, Osaka, Japan) as previously described^69^. C2C12 cells were differentiated in 2% horse serum as previously described^70^. The ON-TARGET plus SMARTpool for mouse GPX4 siRNA (L-072959-01), rat GPX4 siRNA (L-087948-02), rat Acsl4 siRNA (L-091863-02) and a non-targeting pool (siNT, D-001810-10) were purchased from Dharmacon (Lafayette, CO, USA). H9c2 cells were transfected with siRNAs using RNAiMax (Invitrogen) via the reverse-transfection method according to the manufacturer’s protocol.

For primary culture of neonatal rat ventricular myocytes (NRVMs), NRVMs were isolated from 1-day-old Sprague-Dawley rat pups using the Neonatal Cardiomyocyte Isolation System (Worthington biochemical, Lakewood, NJ) according to the manufacturer’s instructions as described previously^71^. NRVMs were plated at a density of 1 million cells on 60-mm dishes coated with 1% gelatine (Corning, Corning, NY, USA) and cultured overnight in DMEM supplemented with 10% FBS, 1% antibiotics, and 0.1 mmol/L BrdU at 37 °C in a humidified incubator with 5% CO_2_. The following day, the cells were placed in serum-free medium without antibiotics for 24 h prior to siRNA transfection. The cells were then transfected with 20 nM siRNA using the DharmaFECT-3 reagent (Dharmacon) according to the manufacturer’s instructions.

For cysteine deprivation, DMEM (no glutamine, no methionine and no cysteine; 21013024, GIBCO) supplemented with 10% FBS and 2 mM L-glutamine (GIBCO) was used because methionine is also a source of cysteine. H9c2 cells and NRVMs were washed in phosphate-buffered saline (PBS) two times, cultured with fresh DMEM or cysteine-deficient DMEM, and incubated for 48 h in the absence or presence of ferroptosis inhibitors.

For glucose deprivation, DMEM (no d-glucose; 11966-025, GIBCO) supplemented with 10% FBS and 1% antibiotics was used.

### Chemicals

RSL3 (S8155), Fer-1 (S7243), birinapant (Biri, S7015), and z-VAD-fmk (zVAD, S7023) were purchased from Selleck Chemicals (Houston, TX, USA). Necrostatin-1 (Nec-1, BML-AP309) was purchased from Enzo Life Sciences (Farmingdale, NY, USA). DMM (136441) and Lip-1 (SML1414) were purchased from Sigma-Aldrich (St. Louis, MO, USA). Recombinant Rat TNF-alpha Protein (TNF-α, 510-RT) was purchased from R & D Systems (Minneapolis, MN, USA).

### Cell viability analysis and PI uptake assay

Cell viability was determined by cellular ATP levels using the CellTiter-Glo reagent according to the manufacturer’s protocol (CellTiter-Glo® 2.0 Assay, G9243, Promega). For the propidium iodide (PI) uptake assay, cells were incubated with 10 μg/mL PI for 15 min and then harvested using trypsin. After washing cells with PBS, the number of dead cells was determined as the PI-positive population using flow cytometry (BD FACSCalibur, BD Biosciences).

### Lipid peroxidation assay

Lipid peroxidation was determined by measuring the amount of malondialdehyde (MDA) using a Lipid Peroxidation assay kit (ab118970, Abcam) according to the manufacturer’s instructions. Briefly, H9c2 cells were homogenized with lysis buffer, and the supernatant was prepared with a thiobarbituric acid (TBA)-glacial acetic acid reagent. After incubation at 95 °C for 1 h, the MDA-TBA adduct was quantified colourimetrically at 532 nm using a spectrophotometer.

### GSH measurements

GSH levels were measured by biochemical determination using Ellman’s reagent. H9c2 cells were washed twice with DPBS and collected into MES buffer using a cell scraper, followed by sonication and centrifugation at 10,000 × *g* and 4 °C for 15 min. Supernatants were collected, and protein was quantified by a Bradford assay. Total GSH was measured using a glutathione assay kit according to the manufacturer’s instructions (Cat# 703002, Cayman Chemical, Ann Arbor, USA) following sample deproteinization using metaphosphoric acid. The GSH level was normalized to the total protein concentration for each sample.

### Western blot analysis

Western blot analysis was performed as described previously^72^. Briefly, cells were lysed in lysis buffer (50 mM Tris-HCl pH 7.5, 150 mM NaCl, 0.5% Triton X-100, and 1 mM EDTA containing a protease inhibitor cocktail). The whole-cell extracts were subjected to western blot analysis using the following antibodies: anti-GPX4 (ab125066, Abcam, Cambridge, UK), anti-Hsp90 (sc-7947, Santa Cruz, CA, USA), anti-ACSL4 (sc-271800, Santa Cruz, USA), anti-β-actin (A5316, Sigma-Aldrich, MO, USA), anti-MLKL (ab196436, Abcam), and anti-PARP (9542, Cell Signaling Technology, Danvers, USA), anti-HIF-1α (14179, Cell Signaling Technology).

### qRT-PCR analysis

Total RNA was extracted with an Easy-spin Total RNA kit (17221, Intron Biotechnology, Korea) according to the manufacturer’s instructions. cDNA was synthesized from 1 μg of total RNA using M-MLV Reverse Transcriptase (Promega) according to the manufacturer's protocol. Amplified cDNA was analysed via real-time PCR (BIO-RAD) with the following primers: mGPX4 (forward), 5′-GCAACCAGTTTGGGAGGCAGGAG-3′; mGPX4 (reverse) 5′-CCTCCATGGGACCATAGCGCTTC-3′; mL32 (forward), 5′-GGCCTCTGGTGAAGCCCAAGATCG-3′; and mL32 (reverse), 5′-CCTCTGGGTTTCCGCCAGTTTCGC-3′.

### Ethics statement

All experimental procedures were performed in accordance with the guidelines and regulations approved by the Animal Care and Use Committee of the Gwangju Institute of Science and Technology (IACUC GIST-2017-006) and Chonnam National University (CNU IACUC-H-2016-36).

## Supplementary information

Supplementary Figure legends

Supplementary Figure

Supplementary table 1

Supplementary table 2-4
