# Supplementary material for: Quantitative proteomic analyses reveal that GPX4 downregulation during myocardial infarction contributes to ferroptosis in cardiomyocytes
Source: Cell Death Dis. 2019 Nov 4;10(11):835. doi: 10.1038/s41419-019-2061-8 (PMC6828761; doi:10.1038/s41419-019-2061-8)
Supplement: Supplementary file 1 — Supplementary Figure legends [file 41419_2019_2061_MOESM1_ESM.docx]

**Supplementary Table 1. Normalized expression of proteins detected by LC/MS-MS at each stage of MI.**

**Supplementary Table 2. Significantly upregulated or downregulated proteins at the early stage (1 day) of MI.**

**Supplementary Table 3. Significantly upregulated or downregulated proteins at the middle stage (1 week) of MI.**

**Supplementary Table 4. Significantly upregulated or downregulated proteins at the late stage (8 weeks) of MI.**

**Supplementary Figure 1. RSL3 induces necrosis-like morphological changes in H9c2 cells**. (a) Cells were treated with various concentrations of RSL3 for 3 h or 5 h and were imaged with a phase contrast microscope (Scale bars, 100 μm). (b) H9c2 cells were incubated in cysteine-depleted DMEM for 36 h and 48 h and were imaged with a phase contrast microscope (Scale bars, 100 μm).

**Supplementary Figure 2. Succinate dehydrogenase and ACSL4 are not involved in RSL3-induced ferroptosis in H9c2 cells.** (a) H9c2 cells pretreated with increasing concentrations of DMM for 3 h were treated with RSL3 as indicated for 24 h. Cell viability was determined by CellTiter-Glo. The data are the mean ± s.d.; n = 3, with *P<0.05, **P < 0.01 and ***P < 0.001 compared to non-treated with a two-sided Student’s t-test. (b) H9c2 cells transfected with ACSL4 siRNA for 48 h were treated with RSL3 for 24 h.

**Supplementary Figure 3.** Differentiation of C2C12 myocytes diminishes ferroptosis sensitivity. (A) RSL3-induced cell death in undifferentiated C2C12 cells. C2C12 cells were treated with increasing concentrations of RSL3 for 24 h. Cell viability was measured using CellTiter-Glo. (B) Inhibition of RSL3-induced death of C2C12 cells by Fer-1. C2C12 cells were treated with 1 μM RSL3 in the presence of 1 μM Fer-1 for 24 h. (C) RSL3-induced cell death in differentiated C2C12 cells. C2C12 cells were differentiated for 5 days and treated with RSL3 as indicated.
