# Supplementary material for: Quantitative proteomic analyses reveal that GPX4 downregulation during myocardial infarction contributes to ferroptosis in cardiomyocytes
Source: Cell Death Dis. 2019 Nov 4;10(11):835. doi: 10.1038/s41419-019-2061-8 (PMC6828761; doi:10.1038/s41419-019-2061-8)
Supplement: Supplementary file 2 — Supplementary Figure [file 41419_2019_2061_MOESM2_ESM.pptx]

## Slide 1
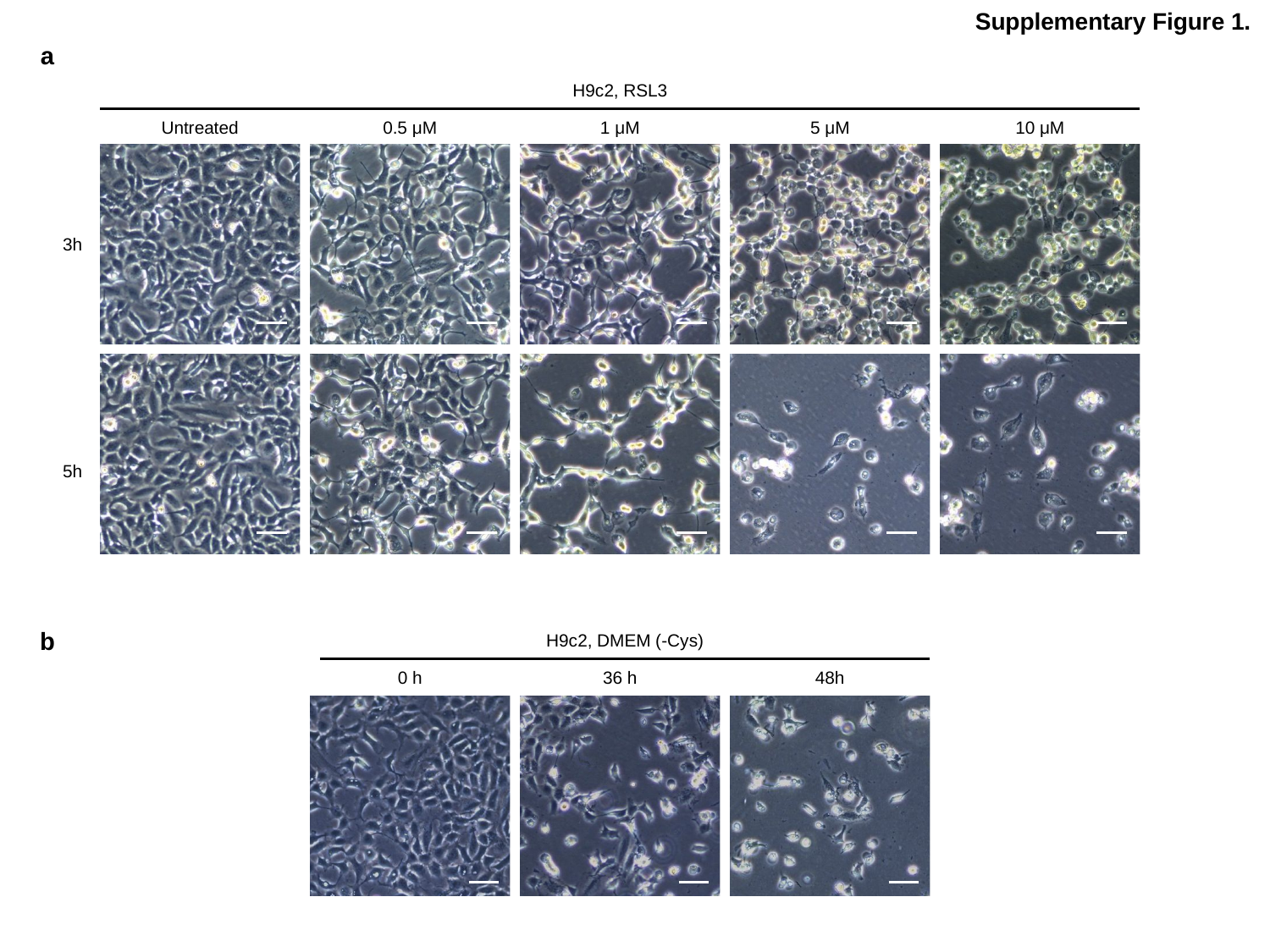

Supplementary Figure 1.
a
H9c2, RSL3
Untreated
0.5 μM
1 μM
5 μM
10 μM
3h
5h
b
H9c2, DMEM (-Cys)
0 h
36 h
48h

## Slide 2
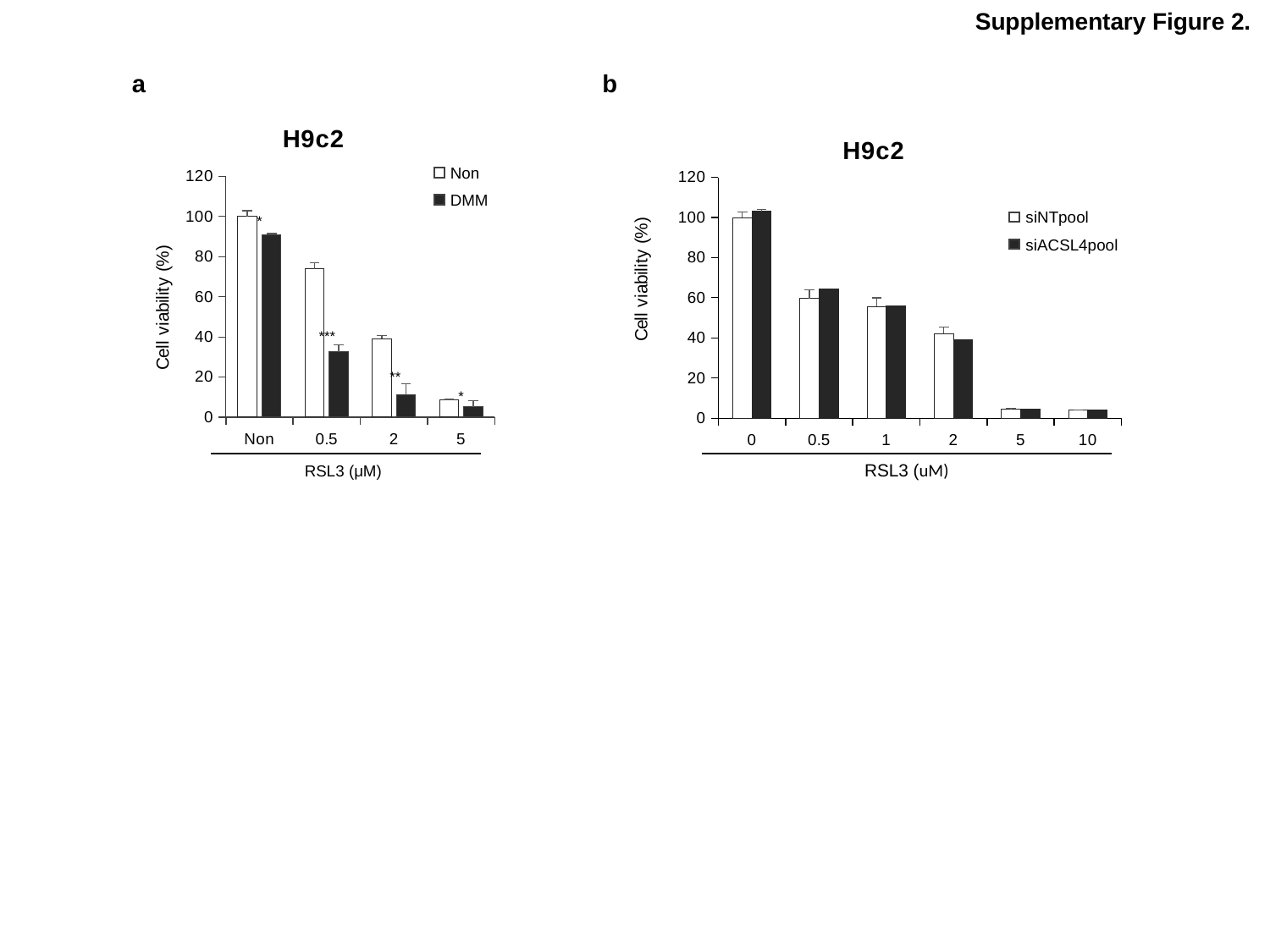

Supplementary Figure 2.
a
b
### Chart: H9c2
| Category | Untreated | 10mM |
|---|---|---|
| Non | 100.0 | 91.0276149347353 |
| 0.5 | 73.95684253715257 | 32.6626749838316 |
| 2 | 39.16772717473696 | 11.005639816688573 |
| 5 | 8.456943372346123 | 5.131190063908651 |Non
DMM
RSL3 (μM)
*
***
**
*
### Chart: H9c2
| Category | siNon | siACSL4 |
|---|---|---|
| 0 | 100.0 | 102.95779970821211 |
| 0.5 | 59.71003365604779 | 64.44630949604452 |
| 1 | 55.699993020243 | 55.87193337566585 |
| 2 | 42.081721037838804 | 38.88414114090087 |
| 5 | 4.531734912233811 | 4.631919960913361 |
| 10 | 4.118822702255483 | 4.340472546572854 |siNTpool
siACSL4pool
RSL3 (uM)

## Slide 3
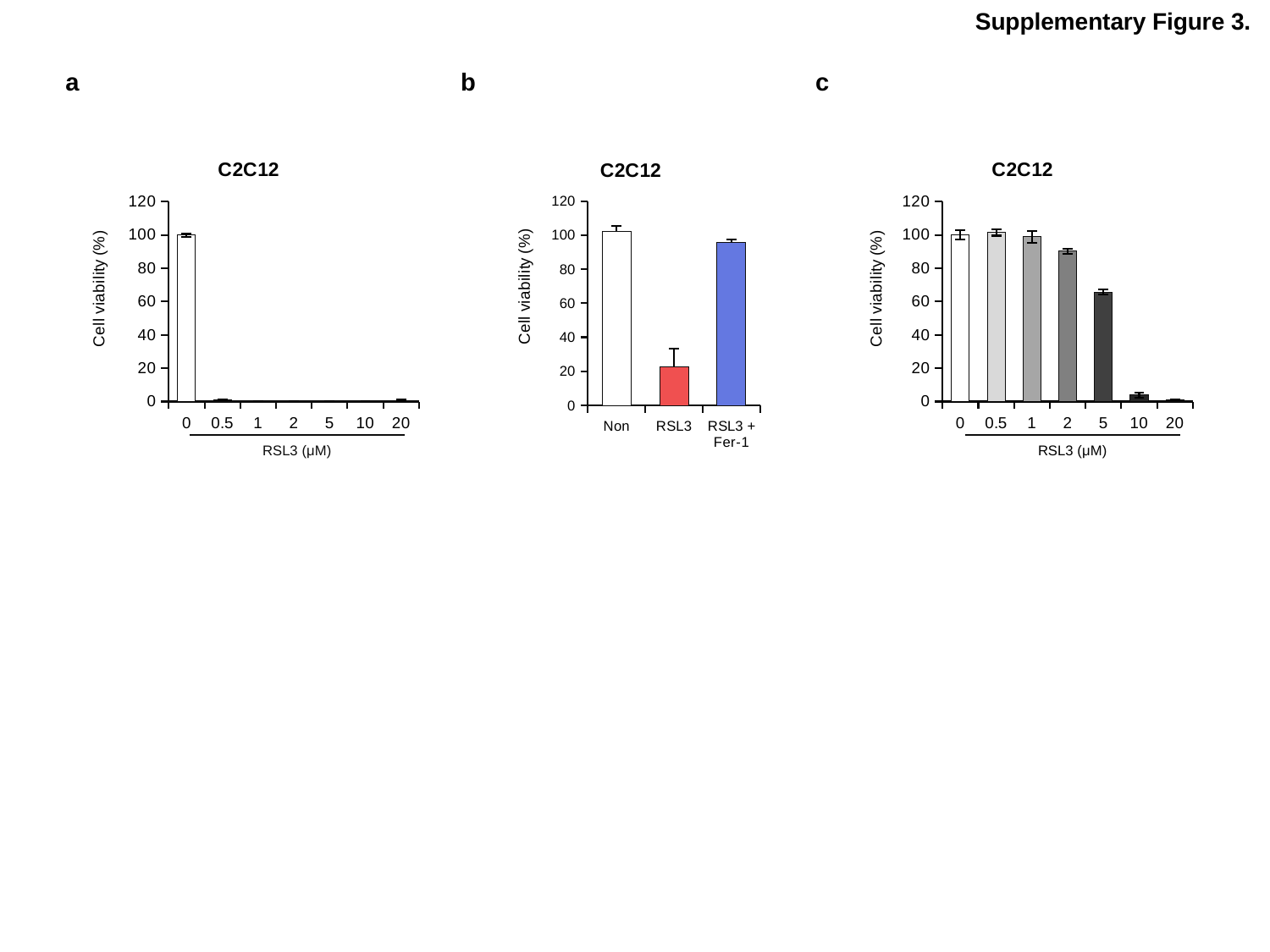

Supplementary Figure 3.
a
b
c
### Chart: C2C12
| Category | |
|---|---|
| 0 | 100.0 |
| 0.5 | 0.7902940645354559 |
| 1 | 0.3730295492372968 |
| 2 | 0.24782779719667905 |
| 5 | 0.2651858218534731 |
| 10 | 0.36351063249002263 |
| 20 | 0.615034009129761 |
### Chart: C2C12
| Category | |
|---|---|
| 0 | 100.0 |
| 0.5 | 101.44018269303074 |
| 1 | 98.84022600760944 |
| 2 | 90.27908690271369 |
| 5 | 65.67388161559411 |
| 10 | 3.9424561674315846 |
| 20 | 0.8829177363822804 |RSL3 (μM)
### Chart: C2C12
| Category | |
|---|---|
| Non | 102.29616656937148 |
| RSL3 | 22.627737626380977 |
| RSL3 + Fer-1 | 95.85105887318463 |RSL3 (μM)
