# Supplementary material for: Quantitative proteomic analyses reveal that GPX4 downregulation during myocardial infarction contributes to ferroptosis in cardiomyocytes
Source: Cell Death Dis. 2019 Nov 4;10(11):835. doi: 10.1038/s41419-019-2061-8 (PMC6828761; doi:10.1038/s41419-019-2061-8)
Supplement: Supplementary file 4 — Supplementary table 2-4 [file 41419_2019_2061_MOESM4_ESM.pptx]

## Slide 1
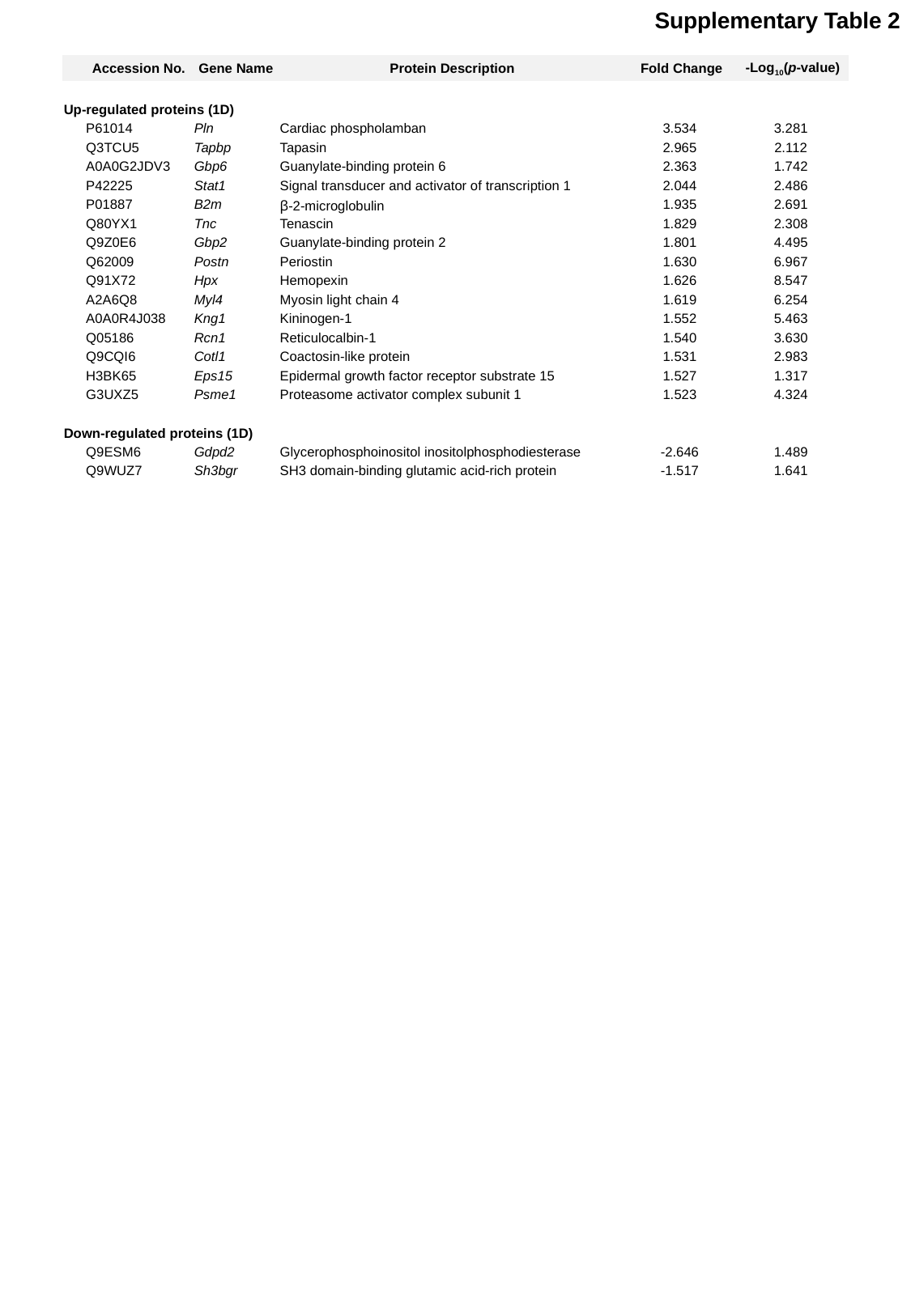

Supplementary Table 2
| | Accession No. | Gene Name | Protein Description | Fold Change | -Log10(p-value) |
| --- | --- | --- | --- | --- | --- |
| | | | | | |
| Up-regulated proteins (1D) | | | | | |
| | P61014 | Pln | Cardiac phospholamban | 3.534 | 3.281 |
| | Q3TCU5 | Tapbp | Tapasin | 2.965 | 2.112 |
| | A0A0G2JDV3 | Gbp6 | Guanylate-binding protein 6 | 2.363 | 1.742 |
| | P42225 | Stat1 | Signal transducer and activator of transcription 1 | 2.044 | 2.486 |
| | P01887 | B2m | β-2-microglobulin | 1.935 | 2.691 |
| | Q80YX1 | Tnc | Tenascin | 1.829 | 2.308 |
| | Q9Z0E6 | Gbp2 | Guanylate-binding protein 2 | 1.801 | 4.495 |
| | Q62009 | Postn | Periostin | 1.630 | 6.967 |
| | Q91X72 | Hpx | Hemopexin | 1.626 | 8.547 |
| | A2A6Q8 | Myl4 | Myosin light chain 4 | 1.619 | 6.254 |
| | A0A0R4J038 | Kng1 | Kininogen-1 | 1.552 | 5.463 |
| | Q05186 | Rcn1 | Reticulocalbin-1 | 1.540 | 3.630 |
| | Q9CQI6 | Cotl1 | Coactosin-like protein | 1.531 | 2.983 |
| | H3BK65 | Eps15 | Epidermal growth factor receptor substrate 15 | 1.527 | 1.317 |
| | G3UXZ5 | Psme1 | Proteasome activator complex subunit 1 | 1.523 | 4.324 |
| | | | | | |
| Down-regulated proteins (1D) | | | | | |
| | Q9ESM6 | Gdpd2 | Glycerophosphoinositol inositolphosphodiesterase | -2.646 | 1.489 |
| | Q9WUZ7 | Sh3bgr | SH3 domain-binding glutamic acid-rich protein | -1.517 | 1.641 |

## Slide 2
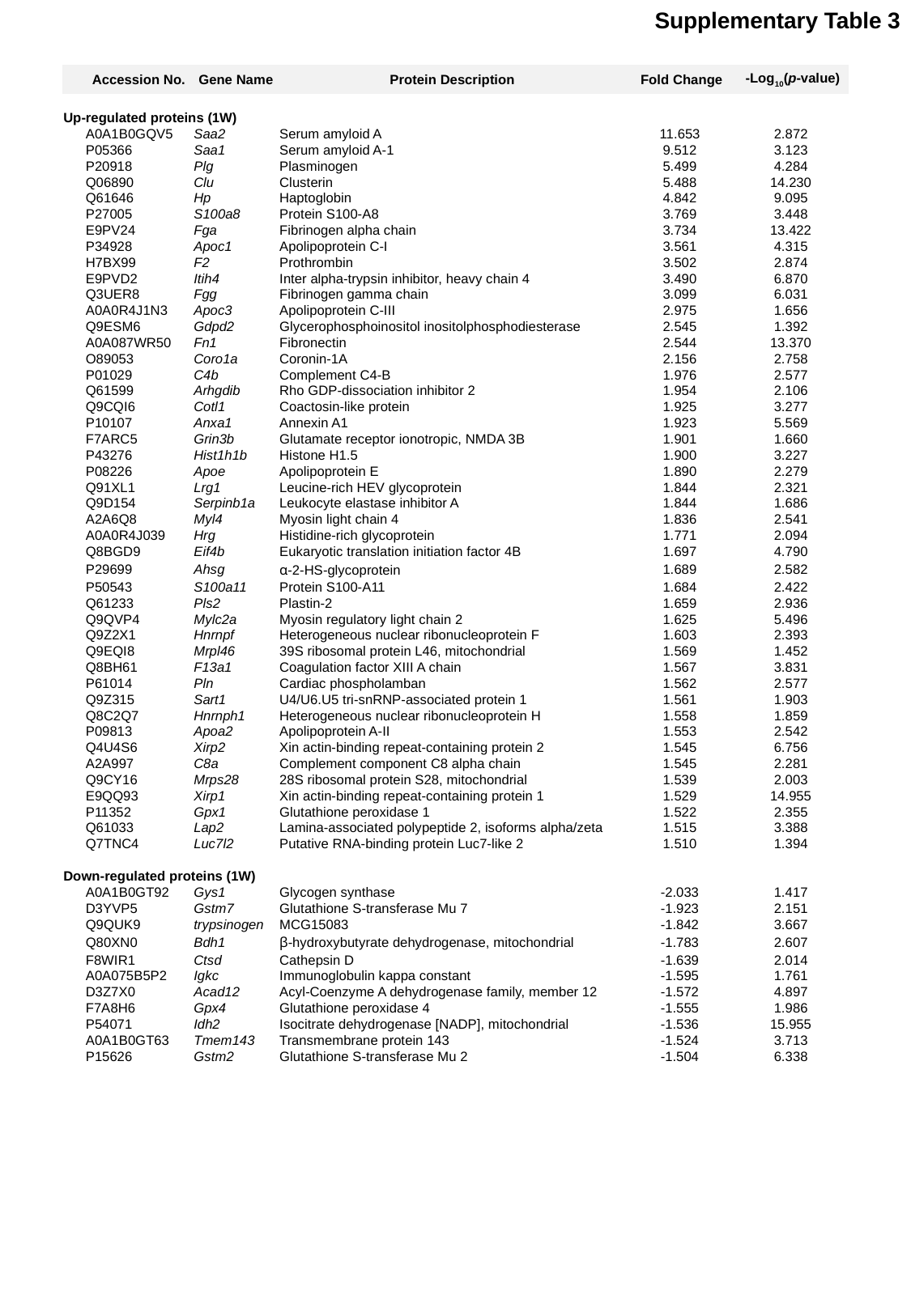

Supplementary Table 3
| | Accession No. | Gene Name | Protein Description | Fold Change | -Log10(p-value) |
| --- | --- | --- | --- | --- | --- |
| | | | | | |
| Up-regulated proteins (1W) | | | | | |
| | A0A1B0GQV5 | Saa2 | Serum amyloid A | 11.653 | 2.872 |
| | P05366 | Saa1 | Serum amyloid A-1 | 9.512 | 3.123 |
| | P20918 | Plg | Plasminogen | 5.499 | 4.284 |
| | Q06890 | Clu | Clusterin | 5.488 | 14.230 |
| | Q61646 | Hp | Haptoglobin | 4.842 | 9.095 |
| | P27005 | S100a8 | Protein S100-A8 | 3.769 | 3.448 |
| | E9PV24 | Fga | Fibrinogen alpha chain | 3.734 | 13.422 |
| | P34928 | Apoc1 | Apolipoprotein C-I | 3.561 | 4.315 |
| | H7BX99 | F2 | Prothrombin | 3.502 | 2.874 |
| | E9PVD2 | Itih4 | Inter alpha-trypsin inhibitor, heavy chain 4 | 3.490 | 6.870 |
| | Q3UER8 | Fgg | Fibrinogen gamma chain | 3.099 | 6.031 |
| | A0A0R4J1N3 | Apoc3 | Apolipoprotein C-III | 2.975 | 1.656 |
| | Q9ESM6 | Gdpd2 | Glycerophosphoinositol inositolphosphodiesterase | 2.545 | 1.392 |
| | A0A087WR50 | Fn1 | Fibronectin | 2.544 | 13.370 |
| | O89053 | Coro1a | Coronin-1A | 2.156 | 2.758 |
| | P01029 | C4b | Complement C4-B | 1.976 | 2.577 |
| | Q61599 | Arhgdib | Rho GDP-dissociation inhibitor 2 | 1.954 | 2.106 |
| | Q9CQI6 | Cotl1 | Coactosin-like protein | 1.925 | 3.277 |
| | P10107 | Anxa1 | Annexin A1 | 1.923 | 5.569 |
| | F7ARC5 | Grin3b | Glutamate receptor ionotropic, NMDA 3B | 1.901 | 1.660 |
| | P43276 | Hist1h1b | Histone H1.5 | 1.900 | 3.227 |
| | P08226 | Apoe | Apolipoprotein E | 1.890 | 2.279 |
| | Q91XL1 | Lrg1 | Leucine-rich HEV glycoprotein | 1.844 | 2.321 |
| | Q9D154 | Serpinb1a | Leukocyte elastase inhibitor A | 1.844 | 1.686 |
| | A2A6Q8 | Myl4 | Myosin light chain 4 | 1.836 | 2.541 |
| | A0A0R4J039 | Hrg | Histidine-rich glycoprotein | 1.771 | 2.094 |
| | Q8BGD9 | Eif4b | Eukaryotic translation initiation factor 4B | 1.697 | 4.790 |
| | P29699 | Ahsg | α-2-HS-glycoprotein | 1.689 | 2.582 |
| | P50543 | S100a11 | Protein S100-A11 | 1.684 | 2.422 |
| | Q61233 | Pls2 | Plastin-2 | 1.659 | 2.936 |
| | Q9QVP4 | Mylc2a | Myosin regulatory light chain 2 | 1.625 | 5.496 |
| | Q9Z2X1 | Hnrnpf | Heterogeneous nuclear ribonucleoprotein F | 1.603 | 2.393 |
| | Q9EQI8 | Mrpl46 | 39S ribosomal protein L46, mitochondrial | 1.569 | 1.452 |
| | Q8BH61 | F13a1 | Coagulation factor XIII A chain | 1.567 | 3.831 |
| | P61014 | Pln | Cardiac phospholamban | 1.562 | 2.577 |
| | Q9Z315 | Sart1 | U4/U6.U5 tri-snRNP-associated protein 1 | 1.561 | 1.903 |
| | Q8C2Q7 | Hnrnph1 | Heterogeneous nuclear ribonucleoprotein H | 1.558 | 1.859 |
| | P09813 | Apoa2 | Apolipoprotein A-II | 1.553 | 2.542 |
| | Q4U4S6 | Xirp2 | Xin actin-binding repeat-containing protein 2 | 1.545 | 6.756 |
| | A2A997 | C8a | Complement component C8 alpha chain | 1.545 | 2.281 |
| | Q9CY16 | Mrps28 | 28S ribosomal protein S28, mitochondrial | 1.539 | 2.003 |
| | E9QQ93 | Xirp1 | Xin actin-binding repeat-containing protein 1 | 1.529 | 14.955 |
| | P11352 | Gpx1 | Glutathione peroxidase 1 | 1.522 | 2.355 |
| | Q61033 | Lap2 | Lamina-associated polypeptide 2, isoforms alpha/zeta | 1.515 | 3.388 |
| | Q7TNC4 | Luc7l2 | Putative RNA-binding protein Luc7-like 2 | 1.510 | 1.394 |
| | | | | | |
| Down-regulated proteins (1W) | | | | | |
| | A0A1B0GT92 | Gys1 | Glycogen synthase | -2.033 | 1.417 |
| | D3YVP5 | Gstm7 | Glutathione S-transferase Mu 7 | -1.923 | 2.151 |
| | Q9QUK9 | trypsinogen | MCG15083 | -1.842 | 3.667 |
| | Q80XN0 | Bdh1 | β-hydroxybutyrate dehydrogenase, mitochondrial | -1.783 | 2.607 |
| | F8WIR1 | Ctsd | Cathepsin D | -1.639 | 2.014 |
| | A0A075B5P2 | Igkc | Immunoglobulin kappa constant | -1.595 | 1.761 |
| | D3Z7X0 | Acad12 | Acyl-Coenzyme A dehydrogenase family, member 12 | -1.572 | 4.897 |
| | F7A8H6 | Gpx4 | Glutathione peroxidase 4 | -1.555 | 1.986 |
| | P54071 | Idh2 | Isocitrate dehydrogenase [NADP], mitochondrial | -1.536 | 15.955 |
| | A0A1B0GT63 | Tmem143 | Transmembrane protein 143 | -1.524 | 3.713 |
| | P15626 | Gstm2 | Glutathione S-transferase Mu 2 | -1.504 | 6.338 |

## Slide 3
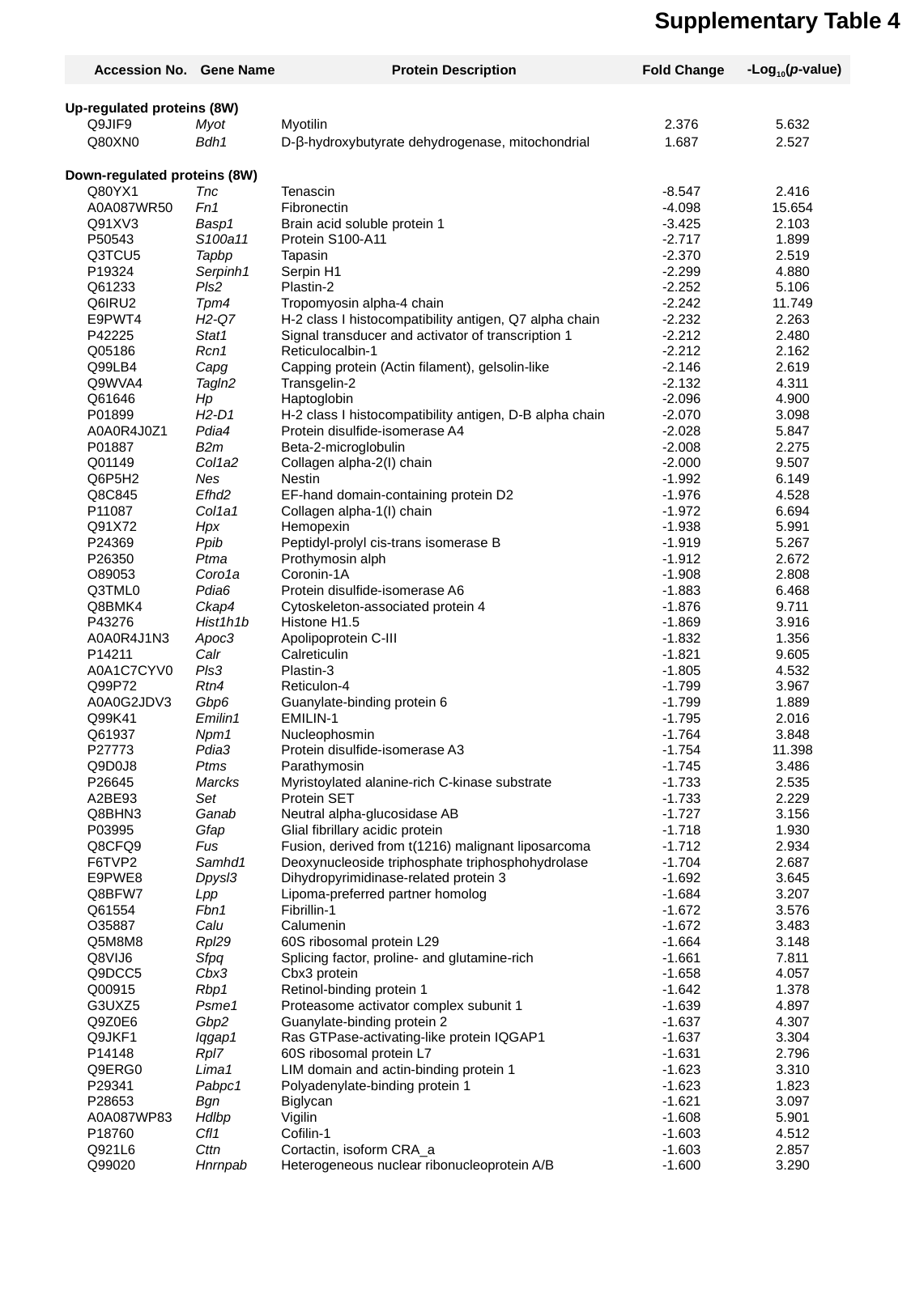

Supplementary Table 4
| | Accession No. | Gene Name | Protein Description | Fold Change | -Log10(p-value) |
| --- | --- | --- | --- | --- | --- |
| | | | | | |
| Up-regulated proteins (8W) | | | | | |
| | Q9JIF9 | Myot | Myotilin | 2.376 | 5.632 |
| | Q80XN0 | Bdh1 | D-β-hydroxybutyrate dehydrogenase, mitochondrial | 1.687 | 2.527 |
| | | | | | |
| Down-regulated proteins (8W) | | | | | |
| | Q80YX1 | Tnc | Tenascin | -8.547 | 2.416 |
| | A0A087WR50 | Fn1 | Fibronectin | -4.098 | 15.654 |
| | Q91XV3 | Basp1 | Brain acid soluble protein 1 | -3.425 | 2.103 |
| | P50543 | S100a11 | Protein S100-A11 | -2.717 | 1.899 |
| | Q3TCU5 | Tapbp | Tapasin | -2.370 | 2.519 |
| | P19324 | Serpinh1 | Serpin H1 | -2.299 | 4.880 |
| | Q61233 | Pls2 | Plastin-2 | -2.252 | 5.106 |
| | Q6IRU2 | Tpm4 | Tropomyosin alpha-4 chain | -2.242 | 11.749 |
| | E9PWT4 | H2-Q7 | H-2 class I histocompatibility antigen, Q7 alpha chain | -2.232 | 2.263 |
| | P42225 | Stat1 | Signal transducer and activator of transcription 1 | -2.212 | 2.480 |
| | Q05186 | Rcn1 | Reticulocalbin-1 | -2.212 | 2.162 |
| | Q99LB4 | Capg | Capping protein (Actin filament), gelsolin-like | -2.146 | 2.619 |
| | Q9WVA4 | Tagln2 | Transgelin-2 | -2.132 | 4.311 |
| | Q61646 | Hp | Haptoglobin | -2.096 | 4.900 |
| | P01899 | H2-D1 | H-2 class I histocompatibility antigen, D-B alpha chain | -2.070 | 3.098 |
| | A0A0R4J0Z1 | Pdia4 | Protein disulfide-isomerase A4 | -2.028 | 5.847 |
| | P01887 | B2m | Beta-2-microglobulin | -2.008 | 2.275 |
| | Q01149 | Col1a2 | Collagen alpha-2(I) chain | -2.000 | 9.507 |
| | Q6P5H2 | Nes | Nestin | -1.992 | 6.149 |
| | Q8C845 | Efhd2 | EF-hand domain-containing protein D2 | -1.976 | 4.528 |
| | P11087 | Col1a1 | Collagen alpha-1(I) chain | -1.972 | 6.694 |
| | Q91X72 | Hpx | Hemopexin | -1.938 | 5.991 |
| | P24369 | Ppib | Peptidyl-prolyl cis-trans isomerase B | -1.919 | 5.267 |
| | P26350 | Ptma | Prothymosin alph | -1.912 | 2.672 |
| | O89053 | Coro1a | Coronin-1A | -1.908 | 2.808 |
| | Q3TML0 | Pdia6 | Protein disulfide-isomerase A6 | -1.883 | 6.468 |
| | Q8BMK4 | Ckap4 | Cytoskeleton-associated protein 4 | -1.876 | 9.711 |
| | P43276 | Hist1h1b | Histone H1.5 | -1.869 | 3.916 |
| | A0A0R4J1N3 | Apoc3 | Apolipoprotein C-III | -1.832 | 1.356 |
| | P14211 | Calr | Calreticulin | -1.821 | 9.605 |
| | A0A1C7CYV0 | Pls3 | Plastin-3 | -1.805 | 4.532 |
| | Q99P72 | Rtn4 | Reticulon-4 | -1.799 | 3.967 |
| | A0A0G2JDV3 | Gbp6 | Guanylate-binding protein 6 | -1.799 | 1.889 |
| | Q99K41 | Emilin1 | EMILIN-1 | -1.795 | 2.016 |
| | Q61937 | Npm1 | Nucleophosmin | -1.764 | 3.848 |
| | P27773 | Pdia3 | Protein disulfide-isomerase A3 | -1.754 | 11.398 |
| | Q9D0J8 | Ptms | Parathymosin | -1.745 | 3.486 |
| | P26645 | Marcks | Myristoylated alanine-rich C-kinase substrate | -1.733 | 2.535 |
| | A2BE93 | Set | Protein SET | -1.733 | 2.229 |
| | Q8BHN3 | Ganab | Neutral alpha-glucosidase AB | -1.727 | 3.156 |
| | P03995 | Gfap | Glial fibrillary acidic protein | -1.718 | 1.930 |
| | Q8CFQ9 | Fus | Fusion, derived from t(1216) malignant liposarcoma | -1.712 | 2.934 |
| | F6TVP2 | Samhd1 | Deoxynucleoside triphosphate triphosphohydrolase | -1.704 | 2.687 |
| | E9PWE8 | Dpysl3 | Dihydropyrimidinase-related protein 3 | -1.692 | 3.645 |
| | Q8BFW7 | Lpp | Lipoma-preferred partner homolog | -1.684 | 3.207 |
| | Q61554 | Fbn1 | Fibrillin-1 | -1.672 | 3.576 |
| | O35887 | Calu | Calumenin | -1.672 | 3.483 |
| | Q5M8M8 | Rpl29 | 60S ribosomal protein L29 | -1.664 | 3.148 |
| | Q8VIJ6 | Sfpq | Splicing factor, proline- and glutamine-rich | -1.661 | 7.811 |
| | Q9DCC5 | Cbx3 | Cbx3 protein | -1.658 | 4.057 |
| | Q00915 | Rbp1 | Retinol-binding protein 1 | -1.642 | 1.378 |
| | G3UXZ5 | Psme1 | Proteasome activator complex subunit 1 | -1.639 | 4.897 |
| | Q9Z0E6 | Gbp2 | Guanylate-binding protein 2 | -1.637 | 4.307 |
| | Q9JKF1 | Iqgap1 | Ras GTPase-activating-like protein IQGAP1 | -1.637 | 3.304 |
| | P14148 | Rpl7 | 60S ribosomal protein L7 | -1.631 | 2.796 |
| | Q9ERG0 | Lima1 | LIM domain and actin-binding protein 1 | -1.623 | 3.310 |
| | P29341 | Pabpc1 | Polyadenylate-binding protein 1 | -1.623 | 1.823 |
| | P28653 | Bgn | Biglycan | -1.621 | 3.097 |
| | A0A087WP83 | Hdlbp | Vigilin | -1.608 | 5.901 |
| | P18760 | Cfl1 | Cofilin-1 | -1.603 | 4.512 |
| | Q921L6 | Cttn | Cortactin, isoform CRA\_a | -1.603 | 2.857 |
| | Q99020 | Hnrnpab | Heterogeneous nuclear ribonucleoprotein A/B | -1.600 | 3.290 |
